# Genomic characterization of *Ensifer aridi*, a proposed new species of nitrogen-fixing rhizobium recovered from Asian, African and American deserts

**DOI:** 10.1186/s12864-016-3447-y

**Published:** 2017-01-14

**Authors:** Antoine Le Quéré, Nisha Tak, Hukam Singh Gehlot, Celine Lavire, Thibault Meyer, David Chapulliot, Sonam Rathi, Ilham Sakrouhi, Guadalupe Rocha, Marine Rohmer, Dany Severac, Abdelkarim Filali-Maltouf, Jose-Antonio Munive

**Affiliations:** 1 Laboratoire de Microbiologie et Biologie Moléculaire, Université Mohammed V, Av Ibn Batouta BP 1014, Rabat, Morocco; 2 IRD, Laboratoire des Symbioses Tropicales et Méditerranéennes UMR113, IRD/INRA/CIRAD/Montpellier SupAgro/Université de Montpellier, TA A82/J, Campus International de Baillarguet, 34398 Montpellier, Cedex 5 France; 3 BNF & Microbial Genomics Lab, Department of Botany, Jai Narain Vyas University, Jodhpur, 342001 India; 4 Université de Lyon, F69622 Lyon, France; 5 CNRS, UMR5557, Ecologie Microbienne and INRA, UMR1418, Université Lyon 1, Villeurbanne, France; 6 Instituto de Ciencias, Benemérita Universidad Autónoma de Puebla, Edif. IC10, Ciudad Universitaria, Col. San Manuel, CP 72570 Puebla, Mexico; 7 Montpellier GenomiX, c/o Institut de Génomique Fonctionnelle, 141 rue de la Cardonille, Montpellier, Cedex 34 094 France

**Keywords:** Rhizobium- legume symbiosis, *Ensifer*, Desert, Adaptation, Comparative genomics

## Abstract

**Background:**

Nitrogen fixing bacteria isolated from hot arid areas in Asia, Africa and America but from diverse leguminous plants have been recently identified as belonging to a possible new species of *Ensifer* (*Sinorhizobium*). In this study, 6 strains belonging to this new clade were compared with *Ensifer* species at the genome-wide level. Their capacities to utilize various carbon sources and to establish a symbiotic interaction with several leguminous plants were examined.

**Results:**

Draft genomes of selected strains isolated from Morocco (Merzouga desert), Mexico (Baja California) as well as from India (Thar desert) were produced. Genome based species delineation tools demonstrated that they belong to a new species of *Ensifer*. Comparison of its core genome with those of *E. meliloti*, *E. medicae* and *E. fredii* enabled the identification of a species conserved gene set. Predicted functions of associated proteins and pathway reconstruction revealed notably the presence of transport systems for octopine/nopaline and inositol phosphates. Phenotypic characterization of this new desert rhizobium species showed that it was capable to utilize malonate, to grow at 48 °C or under high pH while NaCl tolerance levels were comparable to other *Ensifer* species. Analysis of accessory genomes and plasmid profiling demonstrated the presence of large plasmids that varied in size from strain to strain. As symbiotic functions were found in the accessory genomes, the differences in symbiotic interactions between strains may be well related to the difference in plasmid content that could explain the different legumes with which they can develop the symbiosis.

**Conclusions:**

The genomic analysis performed here confirms that the selected rhizobial strains isolated from desert regions in three continents belong to a new species. As until now only recovered from such harsh environment, we propose to name it *Ensifer aridi*. The presented genomic data offers a good basis to explore adaptations and functionalities that enable them to adapt to alkalinity, low water potential, salt and high temperature stresses. Finally, given the original phylogeographic distribution and the different hosts with which it can develop a beneficial symbiotic interaction, *Ensifer aridi* may provide new biotechnological opportunities for degraded land restoration initiatives in the future.

**Electronic supplementary material:**

The online version of this article (doi:10.1186/s12864-016-3447-y) contains supplementary material, which is available to authorized users.

## Background

Global climatic changes affect every continent. Among the most urgent concerns we will have to face in the coming decades, the desertification, which combined to irregular rainfall intensity or frequency and extreme climatic events represent real threats for some local populations. These effects will not only impact cultivable surfaces but also limit areas where humans will be able to live, notably in developing countries where the demographic growth adds on pressure and emergency to react. In the context of sustainable development, the promotion of plants capable of growing in association with symbiotic microorganisms is of particular interest and gain increasing attention. Among these, N-fixing legumes are particularly remarkable as they can develop symbiotic interactions with both bacteria (commonly named rhizobia) and arbuscular mycorrhizal fungi. Overall, grain legumes contain high protein levels that can substitute the extremely high energy and water demanding animal derived protein sources that remain affordable for a great minority of humans. Furthermore, their growth promotion through natural symbioses can reduce fertilizer needs thus bringing economical interest in it while limiting pollution of soils and below ground waters in a context of global warming and clean water resources impoverishment.

With the aim to promote such symbiotic legume growth in a context of global climatic changes, we have to identify symbiotic couples well adapted to a changing environment. This implies both basic and applied advances of researches on symbiosis in relation to environmental constraints. The typical rhizobium-legume symbiosis results from a complex molecular dialogue involving plant and microbial derived compounds that lead to the development of specialized root structures called nodules. In these new organs, bacteria convert atmospheric nitrogen into ammonium which can then be taken up by the plant that in turn provides the bacterium with photosynthesis derived carbohydrates and a protective environment. The comprehension of molecular mechanisms involved in the establishment and functioning of these mutualistic interactions has greatly progressed during the last decade and several independent modes of infection have been identified (for reviews, see [1–3]). Nevertheless, much remains to be done to decipher molecular interplay resulting in efficient symbiotic legume growth in regions subjected to a changing environment (high temperatures, repeated droughts….). Interestingly, with recent advances in sequencing technologies, it is now possible to initiate genome wide studies on any organism that could be viewed as “models”, especially when they can be genetically manipulated. Indeed, taking advantages of advances in the revolutionary next generation sequencing (NGS) technology and of basic knowledge in the symbiosis produced through extensive studies on “historical” model organisms, it is now possible for a small group to study any organism at the genome wide level. A JGI initiative to sequence whole genome of Root Nodule Bacteria (RNB) from different biogeographical regions has led to sequencing of more than 100 RNBs. Such structural genomic database is important for comparative and functional genomics of RNBs [4]. In this context, and by taking advantage of the trapping system which can, through nodule enrichment, rapidly enable isolation of symbiotic bacteria, one can now select rhizobia from any environment of interest and study them at the genome wide level. Using phenotypic tests relative to their nitrogen fixation efficiency, competitivity and plant growth promotion on target legumes, their biofertilizer potential can further be tested upon inoculation which shows that both applied and basic research areas can now be simultaneously approached.

The *Ensifer* (ie. *Sinorhizobium*) genus belongs to the family *Rhizobiaceae* of alpha-proteobacteria and includes a raising number of defined species. These bacteria are rod shaped, gram negative, fast growing, motile, EPS producing and can develop the symbiosis with various legumes. The host range varies however greatly with the species and depends on the symbiotic gene pool present in the microsymbiont which is mostly found on large plasmids called as pSym. While some *Ensifer* strains or species present a rather narrow host range, others have the ability to nodulate a large number of legumes such as the promiscuous rhizobial strain NGR234 that develops the symbiosis with at least 353 legume species, representing 122 genera [5]. There is need to explore such promiscuous strains from legumes growing under harsh environmental conditions. Interestingly, the environment, plant species and physicochemical characteristics of soils can influence the diversity of the symbiotic microbial partners which emphasizes the need to further study rhizobial diversity from heterogeneous environments. Among soil parameters, pH has been shown to play a major role in structuring the rhizobial diversity [6, 7] and appears as a major driver of the bacterial communities [8]. Intriguingly recent studies based on Multi Locus Sequence Analyzes (MLSA) have showed close proximity between *Ensifer* strains isolated previously from desert legume shrubs (*Tephrosia* species) growing in the alkaline soils in the Indian Thar desert [9, 10] with those isolated from Baja California in Mexico (Rocha et al., unpublished data) and Merzouga in Morocco [11] that all share the same type of biome. Surprisingly, these strains were isolated from sandy soils in Baja California where wild bean had been observed (Mexican isolates) or from desert sand dune upon *Acacia* root nodule trapping (Moroccan isolates). Despite the great number of sequences deposited in GenBank databases that were produced from rhizobia diversity studies performed on *Phaseolus* and *Acacia* species, no housekeeping gene sequence identity was found outside this apparent new group of desert rhizobia. Given the presence of isolates sharing the same clade on 3 continents that appear restricted to alkaline hot desert type of biome and the variable hosts with whom they develop effective symbioses, we decided to test whether these strains were indeed belonging to the same and yet undefined species by means of comparative genomic analyzes. Using NGS as well as recent releases of comparative genomic tools and pipelines now enabling the delineation of species from genomic datasets, and rapid prediction of genes and associated functions, we sequenced the genomes of 2 desert rhizobial strains isolated from *Acacia* in Morocco, 2 strains isolated from wild bean in Mexico as well as one *Tephrosia* strain originating from the Indian Thar desert. These 5 genome sequences were combined with the sequence of another Indian Thar desert isolate (TW10) already released [12] and compared to available genomic data from *E. meliloti*, *E. medicae* and *E. fredii*.

## Methods

### Microbiological methods

The list of bacteria used in this study and descriptions of their origin are shown in Table 1. Bacteria were routinely grown at 28 °C for 3–8 days in the media tryptone yeast extract (TY) [13], yeast extract mannitol (YEM) [14] or in the rhizobium minimal medium with succinate as carbon source (RMS) [15]. The strains corresponding to the new species were phenotypically screened using Biolog GN2 plates (Biolog, Inc., Hayward, CA, USA) and compared to the reference strain *Ensifer meliloti* 1021 following manufacturer’s recommendations. Briefly, strains were swabbed from the surface of the TY agar plate, and suspended to a density in IF0 inoculating fluid until a cell density of 52% transmittance on a biology turbidity meter. 150 μl of bacterial suspension were pipetted into each well of the GN2 MicroPlate. The MicroPlates were incubated at 30 °C during 72 h and read using OmniLog™ System. Two repetitions were performed for each strain. HiMedia Discs for carbohydrate fermentation was also tested on the studied strains to compare Biolog results. A total of 16 carbon sources were tested using Andrade’s peptone water following manufacturer’s recommendations. To test malonate utilization, selected strains were grown overnight in TY medium and inoculated in Bioscreen honeycomb 100-well sterile plates at an OD (600 nm) of 0.05 in AT medium [16] supplemented with ammonium sulfate (10 mM) and 40 mM carbon source (either succinate, malonate or a mixture of both at 20 mM each). Cultures were incubated in the dark for 5 days at 28 °C with shaking at medium amplitude. Growth measurements (OD 600 nm) were followed at 20 min intervals using a Microbiology Bioscreen C Reader (Labsystems corp., Finland) according to manufacturer’s instructions. This experiment was performed with five replicates and repeated twice. The isolates were also characterized for their tolerance to high temperatures, sodium chloride (NaCl) and pH as well as for their sensitivity to the antibiotics Chloramphenicol, Gentamycin, Kanamycin, Nalidixic acid, Streptomycin and Spectinomycin using HiMedia antibiotic discs following described methods [17, 18]. Briefly, tolerance to high temperatures were assessed by spotting each bacterial strain onto YEM Agar plates and incubations at temperatures varying from 40 to 48 °C or YEM Agar plates supplemented with different concentrations of NaCl from 0.5 to 3% (*w/v*). The bacterial strains were tested for their tolerance to acidic or alkaline pH using YEM Agar buffered with 20 mM HOMOPIPES (pH 4.5; 5.0), 40 mM MES (pH 5.5; 6.0), 30 mM HEPES (pH 6.8–8.2) and CHES (pH 9.0–10) [19].

**Table 1:** Origin of the strains

Strain name	Country	Area/region	GPS coordinates	Biome type	Soil of origin	Isolation method	Host	Reference
LMR001	Morocco	Merzouga desert	N 31°5′43″/W 3°57′56″	Hot desert	Sand dune without vegetation	Trapping	*Acacia gummifera*	Sakrouhi et al., 2016 [11]
LMR013	Morocco	Merzouga desert	N 31°03′07″/W 3°59′31″	Hot desert	Sand dune without vegetation	Trapping	*Acacia raddiana*	Sakrouhi et al., 2016 [11]
LEM451	Mexico	Baja California	N 22°55′51″/W 109°48′55″	Hot desert	Sand Beach with *Phaseolus filiformis*	Nodule	*Phaseolus filiformis*	Rocha et al., unpublished
LEM457	Mexico	Baja California	N 22°55′51″/W 109°48′55″	Hot desert	Sand Beach with Phaseolus filiformis	Nodule	*Phaseolus filiformis*	Rocha et al., unpublished
TP6	India	Thar desert	N 26°14′49″/E 73°01′16″	Hot desert	Desert soil with Tephrosia	Nodule	*Tephrosia purpurea*	Tak et al., 2016 [10]
TW10	India	Thar desert	N 26°23′55″/E 73°03′46′	Hot desert	Desert soil with Tephrosia	Nodule	*Tephrosia wallichiii*	Gehlot et al., 2012 [12]

### Symbiotic characterization

Selected strains were tested for their ability to produce nodules on their original hosts. Their capacity to form nodules on other wild and cultivated legumes was also investigated using the free-draining pot method described previously [20]. Briefly, strains were tagged with green fluorescent protein (GFP) through triparental mating [21] by transferring pHC60 plasmid carrying the *gfp* gene [22] using the conjugation transfer machinery of helper plasmid pRK2013 [23]. The transformed strains were screened and purified on rhizobia defined medium (RDM) [14] supplemented with the antibiotics tetracycline (10 μg/ml) and streptomycin (100 μg/ml). The GFP-tagged strains were used for inoculating various legume hosts (listed in Additional file 1: Table S1). The control and inoculated plants were maintained in the glass house. After 8 weeks, the inoculated plants were harvested and checked for nodulation. The expression of GFP within nodules was visualized under a fluorescent microscope (Leica DM3000) using an excitation wavelength of 488 nm.

### Genome sequencing, assembly and scaffold ordering

Genomic DNA from 50 ml exponentially growing cell cultures in TY medium was prepared following the “Bacterial genomic DNA isolation using CTAB” procedure described by the JGI [24] except that (i) the Phenol:Chloroform:Isoamyl alcohol (25:24:1) extraction was performed after the RNAse treatment (ii) the high molecular weight DNA was recovered from the last ethanol precipitation step by fishing out the DNA with glass Pasteur pipette. Sequencing libraries were generated using the Illumina TruSeq DNA sample preparation kit (LMR001) or the Nextera DNA sample preparation kit (LMR013, LEM451, LEM457 and TP6) which enabled adaptor ligations of genomic DNA (gDNA) fragments of 700 or 300 bp respectively following manufacturer’s recommendations. Multiplexed libraries were paired-end sequenced by 2 sequential runs of 100 cycles using Hiseq2000 sequencer following Illumina’s recommendations on 1/3 of a flow cell lane for strain LMR001 and 1/4 of a flow cell lane for strains LMR013, LEM451, LEM457 and TP6. Image analysis and base calling were performed using the HiSeq control software and real-time analysis component. Demultiplexing was performed using Illumina’s sequencing analysis software (CASAVA 1.8.2). The quality of the data was assessed using FastQC from the Babraham Institute and the Illumina software SAV (Sequence Analysis Viewer). Potential contaminants were investigated with the FastQ screen software from the Babraham Institute. Genome Assembly was performed with the CLC Genomics workbench software (version 5.5.1, CLC bio) after trimming of adaptor sequences and filtering on low quality scores. Scaffolds above 500 bp with an average coverage above 700×, except for LEM457 for which a significant fraction of the scaffolds which contained notably symbiotic genes showed a mean coverage of 15×, were kept for further analyzes. For each genomic dataset, scaffolds were then ordered using the “Move contigs” tool from the MAUVE aligner program [25] (version 2.3.1) as follows. First scaffolds resulting from the strain LMR001 genome assembly were ordered according to the complete genome of *E. meliloti* 1021 with replicons in the order chromosome, pSymA and pSymB. The reordered scaffolds of LMR001 were subsequently used to order and move scaffolds of the remaining strains LMR013/LEM451/LEM457/TP6 and TW10.

### Quantitative PCR (qPCR) on genomic DNA extracts

To estimate the copy number of the rRNA genes in the 5 newly sequenced isolates, we used *E. meliloti* as a reference whose complete genome (containing 3 copies of the ribosomal RNA genes) is accessible. Primers 16S-870f (5′-GGCAGCAGTGGGGAATATTG-3′) [26] and FGPS505′-313 (5′-GTATTACCGCGGCTGCTG-3′) [27] enabling amplification of a conserved sequence of 162 bp within the 16S rRNA gene were used to estimate its copy number in the genomes of the studied strains by qPCR. For each isolate, genomic DNA was extracted upon growth in YEM medium following a procedure described previously [28]. The genomic DNA extracts were further quantified using the Quant-iT™ PicoGreen kit (Invitrogen™) following manufacturer’s recommendations. The genome copy number used in qPCR assay for 7 dilutions (1/0.2/0.1/0.02/0.01/0.002 and 0.001 ng/μl) and for each strain was estimated based on assembly length for each genome as described previously [29]. The qPCR assays were performed using the LightCyclerR480 system and chemistry (Roche) as recommended by the manufacturer. Efficiencies of the primers (shown in Additional file 1: Table S2) were calculated using the LOG of the calculated genome copy number in each dilution and their respective cycle threshold (Ct) for each compared strain. Given the similar efficiencies of the primer couple used across strains, we could estimate the 16S rDNA copy number (n) in the studied strains by applying for each dilution’s Ct the following equation:

$$ \mathrm{n}=1{0}^{\left(\left(\mathrm{C}\mathrm{t}{\textstyle \hbox{-} }36.985\right)/-3.5101\right)}/\mathrm{N} $$

with −3.5101 and 36.985 corresponding respectively to the slope and intercept of the regression line obtained upon plotting the Ct obtained from *E. meliloti* samples as a function of the logarithm of the 16S copy number in the corresponding dilutions, and N, the calculated genome copy number estimate in the corresponding dilution.

To test whether differences in the predicted symbiotic plasmid copy number could be identified from freshly grown cultures of strains LEM451 and LEM457, the rich YEM medium or RMS complemented or not with the flavonoids Naringenin or Genistein shown to induce nodulation genes in *Phaseolus* rhizobia [30] were used. The copy number of single copy chromosomal genes *gln*II and *rec*A were compared to that of 2 nodulation single copy genes (*nod*C and *nod*F) by qPCR using genomic DNA extracts from these two strains upon growth in these different media. The primer pairs used were recA-F (5′-GCTGAAGTTCTACGCATCGG-3′)/recA-R (5′-AACACCCTCGCCATACATGA-3′), glnII-F (5′-CCACACAGCTTCGTCAACAA-3′)/glnII-R (5′-CGCTGGAAATCGTCTTCAGG-3′), nodC-F (5′-CAAGCCGTTTACTCTCAGCC-3′)/nodC-R (5′-TGAAACAGGGGACGATGACA-3′) and nodF-F (5′-CTATCCACTCCGAGCTCCAG-3′)/nodF-R (5′-CACCGACGTTCTTGAGGTTG-3′). Using these primer pairs, the amplified products were respectively 163, 112, 100 and 138 bp. The qPCR assays were performed as described above. The primer efficiencies were calculated as described for the 16S copy number estimations (Additional file 1: Table S2). For each genomic DNA extract, the copy numbers of the chromosome and of the putative symbiotic plasmids were estimated by averaging data from their respective markers and replicates.

### Plasmid profiles - Eckhardt gels

Plasmid profiles were determined by a modified Eckhardt agarose gel electrophoresis technique as described previously [31, 32]. Briefly, strains were grown in TY medium until the optical density at 600 nm reached 0.6, and 300 μl of culture was used per well. Electrophoresis was carried out at 5 V for 30 min and 85 V for 6 h at 4 °C on a 0.75% agarose gel containing 1% sodium dodecyl sulfate. Plasmid sizes were estimated by comparison with those of *Agrobacterium vitis* S4, *A. fabrum* C58, *E. meliloti* 1021 and *Rhizobium etli* CFN42.

### Genome annotation

Draft genomes consisting in scaffolds ordered upon MAUVE analyzes were integrated into the RAST pipeline and annotated using the “Classic Rast annotation scheme” (FigFams Version 70) [33, 34]. This enabled the recovery of putative Protein Encoding Genes (PEGs), rRNA and tRNAs as well as a functional description of predicted PEGs using “FigFam” classification group of proteins sharing the same function into subsystems allowing rapid identification of specific biological processes. In order to perform intra- and inter-species comparisons and analyze the relative gene content and associated functions, it is important to use the same number of genomes and the same gene prediction and functional assignment tools. Therefore, for *Ensifer* species for which more than 6 genome sequences were available at the time of writing (i.e. *E. meliloti*, *E. medicae* and *E. fredii*), the complete genome sequences corresponding to the reference strains *E. meliloti* 1021, *E. meliloti* Rm41 and *E. medicae* WSM419 as well as draft genomes from other randomly chosen strains [35, 36] were retrieved, integrated into the RAST pipeline and re-annotated using the same method that was utilized for desert rhizobial genomes. Finally, in order to estimate and retrieve core proteome data at the genus level, the draft genome sequences of 4 other *Ensifer* species (*E. arboris*, *E. saheli*, *E. terangae* and *E. sojae*) available were also re-annotated using the same pipeline. The list of genomes used in this study is reported in Table 2.

**Table 2:** Strains used for comparative genomics and general characteristics upon RAST (re)annotation and COG analysis

Strain	Genome					Predicted RNAs (nb)			Predicted Protein Encoding Genes (nb)			Core genome^a^				Accessory genome^a^			
	Accessions	size (bp)	GC (%)	Contig (nb)	Scaffold (nb)	RNA total	tRNA	rRNA (5s, 16S, 23S)	Total	With COG^b^	Without COG^b^	PEG (nb)			GC (%)	PEG (nb)			GC (%)
												Total	With COG^b^	Without COG^b^		Total	With COG^b^	Without COG^b^	
*Ensifer aridi* LMR001	LUAV00000000	6609808	61.7	130	79	53	50	3^d^	6529	4827	1702	5109	4003	1106	62.39	1420	824	596	59.52
*Ensifer aridi* LMR013	LUFU00000000	6789493	61.6	195	185	52	49	3^d^	6743	4893	1850				62.39	1634	890	744	59.33
*Ensifer aridi* LEM451	LUFV00000000	6636281	61.8	131	119	55	52	3^d^	6657	4868	1789				62.39	1548	865	683	59.68
*Ensifer aridi* LEM457	LUFW00000000	6425048	61.8	207	193	56	53	3^d^	6355	4725	1630				62.39	1246	722	524	59.39
*Ensifer aridi* TP6	LUFX00000000	6848280	61.6	173	161	53	50	3^d^	6777	4924	1853				62.39	1668	921	747	59.37
*Ensifer aridi* TW10	AZNX01000000	6802256	61.6	57	57	54	51	1	6787	4922	1865				62.39	1678	919	759	59.31
*Ensifer meliloti* 1021	NC_003037, NC_003047, NC_003078	6691694	62.2	3	3	64	55	3	6657	5142	1515	5733	4525	1208	62.55	924	617	307	59.86
*Ensifer meliloti* Rm41	HE995405-08	7149690	62.1	4	4	64	55	3	7156	nd	nd				62.55	1423	nd	nd	60.01
*Ensifer meliloti* Kh12g	KH12Gv1_ SKH12gv1^c^	6854058	62.2	193	nd	69	57	4	6695	nd	nd				62.55	962	nd	nd	59.87
*Ensifer meliloti* HM007-17	HM007SM17v1_ HM007-17v1^c^	7297325	62.2	141	nd	81	63	6	7149	nd	nd				62.55	1416	nd	nd	60.14
*Ensifer meliloti* M195	M195v1_ SM195v1^c^	7175208	62.1	201	nd	79	61	6	7710	nd	nd				62.55	1977	nd	nd	59.67
*Ensifer meliloti* USDA1002	USDA1002v1_ U1002v1^c^	7568286	62	390	nd	57	51	2	7592	nd	nd				62.55	1859	nd	nd	59.91
*Ensifer medicae* WSM419	NC_009620-22, NC_009636	6817576	61.2	4	4	62	53	3	6930	5098	1832	6031	4647	1384	61.37	899	451	448	59.66
*Ensifer medicae* A321	A321SMEDv1_ SA321v1^c^	7183183	61	236	nd	74	59	5	7297	nd	nd				61.37	1266	nd	nd	58.97
*Ensifer medicae* Kh36d	KH36Dv1_ SKH36dv1^c^	6943414	61.1	185	nd	70	58	4	7062	nd	nd				61.37	1031	nd	nd	59.14
*Ensifer medicae* Kh53a	KH53Av1_ SKH53av1^c^	6889913	61.1	186	nd	60	53	2^e^	7003	nd	nd				61.37	972	nd	nd	58.84
*Ensifer medicae* M1	M1SMEDv1_ SSM1v1^c^	7203177	61.1	260	nd	81	63	6	7319	nd	nd				61.37	1288	nd	nd	59.76
*Ensifer medicae* M22	M22SMEDv1_ SM22v1^c^	7492331	61	235	nd	82	64	6	7662	nd	nd				61.37	1631	nd	nd	59.27
*Ensifer fredii* USDA205	USDA205v1_ U205v1^c^	7177416	62.2	256	nd	66	51	5	6980	5130	1850	5462	4271	1191	62.74	1518	859	659	60.14
*Ensifer fredii* USDA207	USDA207v1_ U207v1^c^	6962831	62.2	311	nd	70	58	2^f^	6791	nd	nd				62.74	1329	nd	nd	60.13
*Ensifer fredii* CCBAU05557	AJQM00000000	6587698	62.3	382	nd	54	51	1	6386	nd	nd				62.74	924	nd	nd	59.98
*Ensifer fredii* CCBAU45436	AJQO00000000	6815462	62.2	327	nd	68	56	4	6594	nd	nd				62.74	1132	nd	nd	59.95
*Ensifer fredii* CCBAU83643	AJQQ00000000	6876949	62	438	nd	50	47	1	6722	nd	nd				62.74	1260	nd	nd	59.43
*Ensifer fredii* CCBAU83753	AJQS00000000	6832812	62.1	417	nd	52	49	1	6728	nd	nd				62.74	1266	nd	nd	59.74
*Ensifer arboris* LMG14919	NZ_ATYB0100000000.1	6850303	62	16	nd	62	53	3	6670	nd	nd	na	na	na	na	na	na	na	na
*Ensifer saheli* USDA4893	USDA4893v1_ U4893v1^c^	6179454	63.5	203	nd	61	52	1^g^	5899	nd	nd	na	na	na	na	na	na	na	na
*Ensifer terangae* USDA4894	USDA4894v1_ U4894v1^c^	7095078	61.3	140	nd	79	61	6	7006	nd	nd	na	na	na	na	na	na	na	na
*Ensifer sojae* CCBAU05684	AJQT00000000.1	5964336	62	231	nd	50	47	1	5810	nd	nd	na	na	na	na	na	na	na	na

*nd* not determined

*na* not applicable

^a^Based on 6 genomes/species (Ozer et al. 2014 [37])

^b^COG prediction by RPSBlast using e-value of 0.001 cutoff

^c^Accessions from MaGe (https://www.genoscope.cns.fr/agc/microscope/home/index.php)

^d^Estimated based on quantitative PCR

^e^An extra copy of the 16S rRNA was identified in the genome

^f^3 extra copies of the 5S and of the 23S rRNAs were identified in the genome

^g^3 extra copies of the 16S and of the 23S rRNA were identified in the genome

### Estimation and comparison of the Core and Pan-genomes in 4 *Ensifer* species

Once (re)-annotated, the 6 genomes for each species (*E. aridi, E. meliloti, E. medicae* and *E. fredii*) were used to estimate their respective core species genomes. For this purpose, we used the SPINE tool recently developed by Ozer and colleagues [37] with options set to 100% of input genomes, 85 minimal percent identities of nucmer alignments. The order of genomes used for *E. aridi* was LMR001, LEM451, TP6, LMR013, LEM457 and TW10; for *E. meliloti*, we used the strain order 1021, Kh12g, Rm41, HM007-17, M195 and USDA1002; for *E. medicae*, the order was WSM419, A321, Kh36d, M1, Kh53a and M22 and finally for *E. fredii*, the strain order was USDA205, CCBAU0557, CCBAU45436, USDA207, CCBAU83643 and CCBAU83753. For each species compared, the proportions of core/accessory genomes were retrieved and the PEG list included in the conserved segments and corresponding to the reference genome (first genome in the above lists) was retrieved and considered as the species conserved PEG fractions or the core species genome.

To estimate the core genome at the *Ensifer* genus level, the LMR001 Bidirectional Best Hits (BBH) of translated proteins showing more than 70% identity for all 28 strains belonging to 8 *Ensifer* species (Table 2) were retained and retrieved. To identify genes and/or functions that were present in all 6 strains in an *Ensifer* species but not conserved at the genus level, the fraction of PEGs corresponding to the core genus level were subtracted from each core species genome.

For each one of the 4 compared *Ensifer* species, a pan-genome analysis was also performed using Blast analyzes as implemented in the GView package [38] with as cutoffs, 1e-10 e-value, 80% identity and 80% of minimal alignment length.

### Functional description and comparison of 4 *Ensifer* species

To get insights in functions associated to desert symbiotic bacteria, Clusters of Orthologous Groups of proteins (COG) numbers were identified by Reverse Position Specific Blast (RPS Blast) against the COG database [39] using the WebMGA server [40]. COG number(s) with e-values below 0.001 and their respective functional class and category were retrieved for all predicted PEGs and the 4 core species genomes. Furthermore, the Kyoto Encyclopedia of Genes and Genomes (KEGG) resources [41] were also used to assign KEGG orthology (KO) identifiers from core proteomes, reconstruct pathways and modules to gain insights on species conserved functional units. Finally, prophage and insertions sequences (IS) were predicted from pseudo-assembled genomes using the PHAST [42] and ISsaga [43].

### Genome based species delineation and phylogeny

To test whether the 6 desert strains sequenced belong to the same species, the Average Nucleotide Identity (ANI) [44], *in silico* based DNA-DNA Hybridization (DDH) estimates [45] and correlations between tetranucleotide signatures using JSpecies [46, 47] were calculated from draft genome sequences for all pair-wise comparisons [46]. For the ANI calculation, alignment options used were 70% minimal identity over more than 700 bp in fragment windows of 1000 bp with a 200 bp step size on reciprocal best hits (two-way ANI). For the DDH estimation, we used the Genome to Genome Distance Calculator (GGDC) with the BLAST+ alignment method which uses the Generalized Linear Model with the recommended formula (identities/High-scoring Segment Pair lengths). The resulting probability that DDH > 70% was calculated by logistic regression as described by Meier-Kolthoff and colleagues [45].

The phylogenetic positioning of the desert *Ensifer* strains among alpha-rhizobia was analyzed by Neighbor Joining (NJ) using 103 concatenated genes from various strains, species and genera (listed in Additional file 1: Table S3a) that showed BBH against the LMR001 proteome and which presented a minimum of 70% identity over at least 80% of the protein length and for which the corresponding nucleotide sequences could be aligned manually. These 103 genes could be considered as the most conserved both at the nucleotide and protein levels among compared organisms. The list of strains and species as well as the gene names corresponding to the well annotated genome of *E. meliloti* 1021 are reported in the (Additional file 1 Tables S3a and S3b). Predicted gene sequences were retrieved for all strains and species, aligned using ClustalX and concatenated. Complete deletions were performed at gap locations. The NJ consensus tree was inferred using the Kimura 2-parameter model and 1000 replications.

To increase the resolution of the evolutionary relationships between the 6 strains belonging to the new *Ensifer* species, phylogenies were also performed using these 103 alpha-rhizobia conserved concatenated gene sequences which were in great majority (100/103) located in contigs showing synteny with *E. meliloti* chromosome. Furthermore, to compare evolutionary relationships according to replicon type synteny with *E meliloti* genome, a selection of *E. aridi* core genes which were all located in contigs ordered according to *E. meliloti* 1021 symbiotic plasmid pSymA (86 genes) or the chromid pSymB (55 genes selected according to their distribution along the contigs in synteny with *E. meliloti* 1021 chromid) were retrieved for all 6 strains. The Additional file 1 (Table S4) reports *E. aridi* PEG lists corresponding to the 103 alpha-rhizobia most conserved gene set (Table S4a), the 86 pSymA conserved orthologous gene set (Table S4b) and the selected 55 pSymB conserved orthologous gene set (Table S4c). These PEGs were retrieved, aligned and phylogenies were inferred as described above.

## Results and discussion

### Origin of the strains

By crossing data obtained from MLSA based diversity studies on rhizobia recovered from hot deserts in Asia [9, 10, 48], Africa [11] and America (Rocha et al., unpublished data), a possibly new species within the *Ensifer* genus was suggested. Recovered exclusively from sandy alkaline soils in hot arid areas, this new group of symbiotic bacteria has so far never been sampled in less perturbed environments suggesting a particular adaptation to such biome. To verify if these isolates belong to the same *Ensifer* species and to estimate their genomic and phenotypic specificity, 6 isolates originating from the 3 continents were selected (Table 1). Two Indian strains isolated from nodules of *Tephrosia purpurea* (TP6) and *T. wallichii* (TW10), two Mexican strains (LEM451 and LEM457) nodulating wild bean (*Phaseolus filiformis*) growing occasionally on sand beaches in southern Baja California (Mexico) as well as 2 strains (LMR001 and LMR013) nodulating *Acacia gummifera* and *A. raddiana* (also called *Vachellia gummifera* and *V. tortillis* respectively) isolated by trapping using sand dune from the Merzouga desert (Morocco) were chosen. Except TW10 for which the draft genome was published previously, the genomes of the 5 other strains were sequenced.

### Genome sequencing, assembly and scaffold ordering

The draft genomes of the 5 strains LMR001, LMR013, LEM451, LEM457 and TP6 were obtained using the Illumina technology (Hiseq2000) and a paired-end sequencing approach consisting of 2 sequential runs of 100 cycles for each strain. The number of sequences produced that passed the quality filter is reported in Additional file 1 (Table S5). They varied from 34 to 71 million depending on the library and corresponded at least to 75% of the total number of sequences produced prior filtering. Sequences that passed the quality filter were trimmed and subsequently assembled with the CLC Genomics workbench. The results of the assembly are represented in Additional file 1 (Table S5). The genome sizes of the studied strains varied from 6.4 to 6.8 Million base pairs (Mbp) and were in the range of other *Ensifer* genomes. The Whole Genome Shotgun (WGS) projects for strains LMR001, LMR013, LEM451, LEM457 and TP6 have been deposited at DDBJ/ENA/GenBank under the accessions LUAV00000000, LUFU00000000-LUFX00000000 respectively (Table 2) and the corresponding versions described in this paper are versions LUAV01000000, LUFU01000000-LUFX01000000. The number of scaffolds in the newly sequenced strains varied from 79 to 193. Surprisingly, for the strain LEM457, a set of 111 scaffolds corresponding to approximately 573 kilo base pairs (Kbp) in length showed a significantly lower sequencing coverage mean of 15.8 with a Standard Deviation (SD) of 3.4 compared to the remaining scaffolds which presented a mean coverage 2 log higher 2467.3 (SD of 1842.8). Moreover, these low coverage scaffolds were containing many known symbiotic genes that were missing in the remaining scaffolds and were therefore kept for downstream analyzes. The N50 values varied between 175 and 367 Kbp with L50 values ranging from 7 to 11 in the newly sequenced strains.

**Fig. 1:**
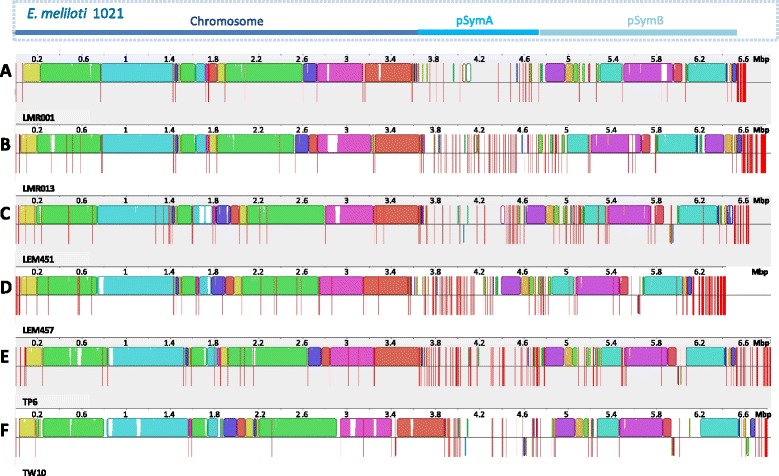
Genome wide alignment of the 6 desert rhizobial draft genomes. The finished genome of *Ensifer meliloti* 1021 consisting of concatenated replicons in the order chromosome, pSymA and pSymB (indicated on the *top* of the figure) was first used as a reference to order the 79 scaffolds of LMR001 (**a**) using the progressive MAUVE aligner. The resulting reordered pseudo-assembled LMR001 genome was subsequently used as reference to reorder the scaffolds of LMR013 (**b**), LEM451 (**c**), LEM457 (**d**), TP6 (**e**) and TW10 (**f**). *Scales* above each map refer to genome size in million base pairs (Mbp). Scaffold borders are indicated by *vertical red bars* and locally collinear blocks are shown using similar colors across draft genomes. *Colorless spaces* indicate accessory genomic regions

The alignment of conserved regions from the 6 genomes corresponding to the newly identified species upon scaffold ordering is shown in Fig. 1. Globally, we found that large conserved collinear blocks were found for the regions showing similarities with the *E. meliloti* 1021 chromosome as well as the chromid pSymB while a central region and the 3′ end of genomes showed fewer similarities between compared strains. In *Ensifer* strains and species for which complete genomes are available the symbiotic genes are often located in large plasmids. Based on this knowledge, we suggest that the central variable region and probably part of the 3′ end of assembled genomes in our new species are located on symbiotic plasmids. For instance, *nod* and *nif* genes involved in symbiosis development and functioning as well as *rep*, *tra* and *trb* gene clusters involved in plasmid replication and transfer were identified in scaffolds located in these variable regions for all compared strains. Furthermore, when we compared the actual location of the genomic fraction that represented the low covered scaffolds from strain LEM457 (data not shown), we found that these mapped to the central region showing similarities to pSymA and the 3′ end of the pseudo-assembled genome which include scaffolds with lowest similarities as they were neither conserved at the genus nor at the species levels (data not shown). However, the current drafts do not allow identifying the actual replicon type (chromosome, chromid and plasmid(s)), number and size that would require an additional gap closure sequencing strategy, pulse field gel electrophoreses or the use of a promising sequencing technology that produces very long reads such as PacBIO. However, the results of the Eckhardt gel analysis clearly showed the presence of extra-chromosomal replicons in all studied isolates (Additional file 2: Figure S1). Notably, it demonstrates the presence of large plasmids whose range varied greatly between compared strains. Interestingly, a large replicon of approximately 1.6 Mbp could be identified in all desert strains which may correspond to a large plasmid (or chromid) and additional replicons of variable sizes (ranging approximately from 0.1 to 1.4 Mbp) were predicted in the newly sequenced strains. The Moroccan strains presented 2 additional replicons each including a large one of ca 1.3 Mbp and a smaller one of ca 200 Kbp (LMR001) or 400 Kbp (LMR013). The profile of one of the Mexican strain LEM451 also indicated the presence of 2 additional replicons (of ca 800 and 250 Kbp), while the profile obtained from the second strain (LEM457) showed the presence of a single plasmid of ca 100 Kbp. Finally, as for the Moroccan strains, the Indian strain TP6 profile indicated the presence of 2 extra replicons of ca 1.3 and 0.5 Mbp (Additional file 2: Figure S1). Intriguingly, the strain LEM457 appeared to lack a replicon as compared to the other strains. The net difference in sequence coverage obtained between the two sets of scaffolds upon mapping of sequencing reads for this particular strain and the lack of an observable replicon by Eckhardt gel electrophoresis when compared to other strains strongly suggest a partial loss of a replicon which could correspond, based on the presence of symbiotic genes in low coverage scaffolds, to a symbiotic plasmid. Such loss or instability of accessory plasmid by this symbiotic bacterium may result from subculturing without interaction with its host plant [49]. To test whether we could show such plasmid loss or instability, a qPCR assay was performed using genomic DNA extracts from fresh cultures of the two strains LEM451 and LEM457 isolated from the same host (*Phaseolus filiformis*) and which share a set of 329 orthologs (BBH using 50% identity as cutoff) located in the low coverage scaffolds of LEM457. Two chromosomal single copy genes (*recA* and *glnII*) as well as two conserved symbiotic markers (*nodC* and *nodF*) were used as targets. The results shown in Additional file 2: Figure S2 clearly demonstrates that regardless the growth medium used, gDNA extracts from strain LEM457 were deprived of the targeted nodulation genes as no amplification was observed which contrast with results obtained from strain LEM451. This loss most probably results from the selection of clones that had already lost the plasmid for the qPCR assays but corroborate results obtained from Eckhardt gel analysis. Nevertheless, the loss of the symbiotic plasmid may be of interest in biotechnology through the introduction of other plasmids that may broaden the host spectrum of this species but should however also be taken into account for a potential use as biofertilizer (inoculants). The instability of this putative symbiotic plasmid was only observed in the strain LEM457. Here, the main objectives were to clarify whether the desert strains originating from the three continents belonged to the same new species within the *Ensifer* genus and identifying conserved gene fraction and putative functions associated with this possibly new taxon.

### Genome automated annotations

The pseudo-assembled genomes of the desert strains studied were integrated into the RAST pipeline that enabled the prediction of PEGs and RNA features for all strains. The results of the automated annotation by RAST are shown in Table 2 and the genomes can be browsed from RAST website [50]. The number of PEGs varied from 6355 to 6787 which corresponded to an approximate density of 1 gene every Kbp of sequence which is comparable to other *Ensifer* strains and species (see Table 2). The number of predicted RNAs varied from 52 to 56 and only one copy of the rRNAs (5S, 16S and 23S) was predicted upon assembly in the newly sequenced strains. However, the fact that in the 5 newly sequenced strains, the rRNA genes were assembled into a single scaffold of approximately 6.2 Kbp (encompassing the 16S, 23S and 5S rRNAs as well as 3 tRNAs) with a greater sequencing coverage when compared to the sequencing read coverage at the genome wide level suggests that this locus is repeated. Indeed, the copy number of ribosomal RNA genes may be underestimated due to assembly limitations of large repeated regions when using the Illumina sequencing technology. To clarify this, a qPCR assay was performed (see Methods for details). Using *E. meliloti* as a reference, the calculation in the studied genomes returned a copy number ranging from 3.26 to 3.36 (with SD < 7%) except for strain LEM457 which indicated a copy number of 3.67 (with 7% of SD). The fact that our calculation did not return integers was expected as the calculation was performed assuming that (i) the molecular weight of the genomic DNA was correlated to the genome size regardless of GC % differences between the genomes and (ii) there is one copy of each genomic replicon per cell. The difference obtained for the strain LEM457 may result from the loss of the symbiotic plasmid representing a tenth of the remaining genome size in that particular strain gDNA extract which would higher the 16S rDNA copy number estimate by 10%. Nevertheless, these results suggest that the scaffold which contained all the ribosomal RNA genes was present in multiple copies, most probably 3, in the genomes under study. The GC content varied from 61.6 to 61.8%. The results of the Genome annotation obtained for the 22 other *Ensifer* strains and species are also shown in Table 2. The GC % was the most conserved feature at the species level.

### Genome based species delineation and positioning of the strains in the phylogeny of alpha-rhizobia

**Fig. 2:**
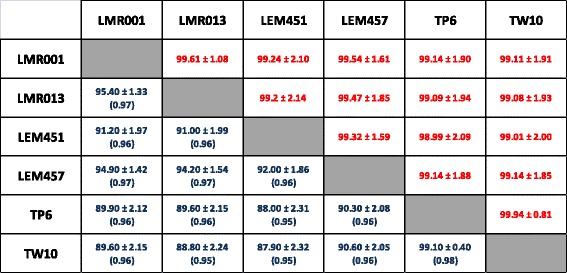
Genome wide based species delineation among desert *Ensifer* strains. *Top right* values on the matrix (*red*) show results obtained from a two-way ANI calculation expressed as a % ± Standard Deviation while *Bottom left* values on the matrix (*blue*) show results from DDH estimations using Generalized Linear Model and the recommended formula (Identities/High Scoring Pairs) and are expressed as a % ± Standard Deviation together with the probabilities that DDH > 70% (i.e., same species) in *parentheses*

One of the objectives of the present study was to verify whether the desert *Ensifer* strains isolated from the three continents belong to a new *Ensifer* species. Using draft genome sequences from the 6 strains, the ANI and *in silico* DDH estimations were calculated for all pair-wise comparisons. The results of the comparisons are summarized as a matrix in the Fig. 2 and show clearly that, regardless of the strain or the method, all 6 strains could be regarded as belonging to the same species. The distributions of the % identities of nucleotide fragments used for the ANI calculation are represented as box plots in Additional file 2: Figure S3. The number of fragments used for the ANI measures (ie. showing at least 70% identity over 1 Kbp) ranged from 24,801 for the most divergent strains (TW10 vs LEM451) to 30,794 for the closest strains (TW10 vs TP6). More than 75% of the 1000 bp fragments were at least 99% identical in all comparisons suggesting high sequence conservation among strains, regardless of their origin. In line with these results, using the TETRA analysis tool from JSpecies, the regression coefficients from pair-wise comparisons of tetranucleotide signatures were very high (above 0.9996, data not shown). Intra- and inter-species ANI calculations were also performed using available genome sequences confirming that the new taxon described here corresponds to a new species with ANI values comparable to that of *E. meliloti*, *E. medicae* or *E. fredii* intra-species comparisons and which differ greatly from inter-species ANI values that were all below 95% which corresponds to the current cutoff value used for species delineation (data not shown). With the reduction of WGS sequencing costs, such methods will be increasingly used in the future and may replace the traditional and tedious DNA-DNA hybridization approach used for species delineation. Furthermore, the availability of genomic data and comparative tools may help better understanding the evolution and specificities of each species through comparative genomics [51].

**Fig. 3:**
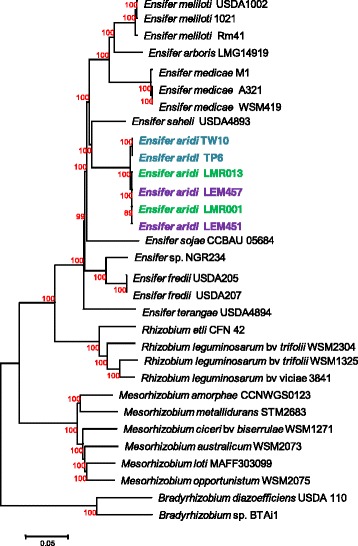
Neighbor Joining tree showing phylogenetic relationships among 31 rhizobial strains. The tree was computed using 103 conserved gene sequences that were concatenated and aligned. There were 114,534 aligned bases in the final dataset after removal of ambiguous positions. Evolutionary distances were computed using the K2P method and are in the units of the number of base substitutions per site. The percentage of replicate trees in which the associated taxa clustered together in the bootstrap test (1000 replicates) is shown next to the branches (*red*). The tree is drawn to scale, with branch lengths in the same units as those of the evolutionary distances used to infer the phylogenetic tree. Moroccan, Mexican and Indian strains are highlighted in *green*, *purple* and *blue* respectively

To evaluate the phylogenetic positioning of the new *Ensifer* species among selected alpha-rhizobia, 103 of the most conserved genes among alpha-rhizobia (genera *Rhizobium*, *Mesorhizobium*, *Ensifer* and *Bradyrhizobium*) were selected, aligned and concatenated. Merged sequences were analyzed by Neighbor Joining and the resulting tree from 1000 replications is shown in Fig. 3. Again, all the 6 strains clustered together and showed very little sequence divergence as indicated by the low branch length within the clade they formed which was comparable to that found between strains belonging to the same species such as *E. meliloti* or *E. medicae*. We obtained bootstrap values of 100% for nearly all nodes within the *Ensifer* clade showing the robustness of the tree generated. The closest relative of the presently described species was *E. saheli*, a species recovered from *Sesbania* nodule from arid hot environment.

Given the isolation of this symbiotic *Ensifer* species in arid and hot environments from three continents, we here propose the name *Ensifer aridi*. This species may have acquired locally symbiotic and accessory genes most probably in the form of large (symbiotic) plasmids which could explain the differences of hosts with which they associate and consequently symbiovars may further be defined [52].

### Comparative genomics

**Fig. 4:**
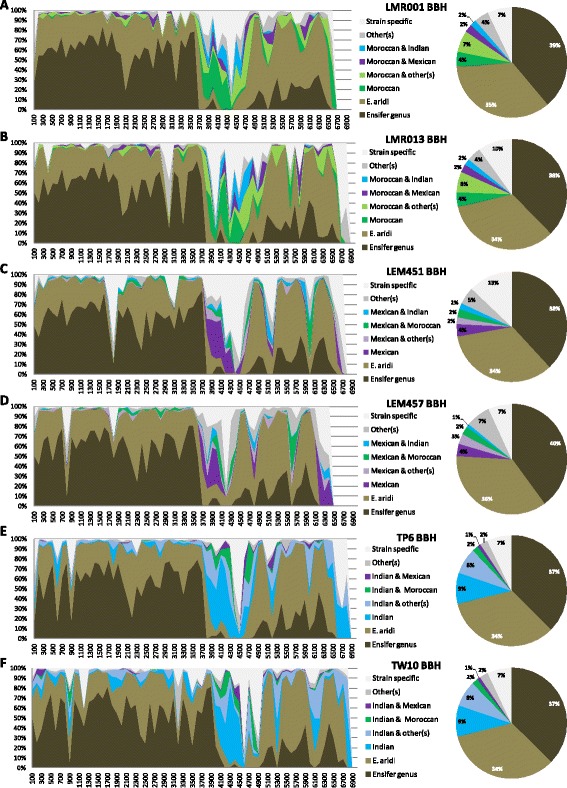
Proportion of Bidirectional Best Hits (BBH) along pseudo-assembled *Ensifer aridi* genomes in relation to their occurrence in all *Ensifer* strains (28) and species (8) compared (*Ensifer* genus conserved), all *E. aridi* strains presently compared (6, *E. aridi* conserved), in *E. aridi* strains originating from the same geographical location (Morocco, Mexico or India). The 100% piled area graphs on the *left panel* show the percentage of predicted PEGs in each category along pseudo-assembled genomes of *Ensifer aridi* strains LMR001 (**a**) and LMR013 (**b**) originating from Morocco, LEM451 (**c**) and LEM457 (**d**) originating from Mexico, TP6 (**e**) and TW10 (**f**) originating from India. PEGs numbers being ordered according to their genomic location on the pseudo-assembled genomes, we used a 100 PEGs step which roughly correspond to 100 Kbp increments from which the % of PEGs in each class was calculated. The pie charts on the *right side* of each area graph show the global repartition of hits in defined categories for each strain using the same color codes. Moroccan, Mexican and Indian indicates presence of hits in both strains from the same geographical origin (respectively LMR001 and LMR013, LEM451 and LEM457 or TP6 and TW10) while “Other(s)” refers to hits in strains from another geographical origin but without geographical conservation

To compare the 6 studied desert *Ensifer* genomes, the predicted PEGs were classified along the pseudo-assembled draft genomes according to their presence in (i) all *Ensifer* strains compared (Core genus genome including 8 species and 28 strains), (ii) all the 6 desert strains but not all other species (Species conserved), (iii) strains from the same geographical origin (Indian, Moroccan or Mexican) (iv) several strains but without geographical conservation or (v) uniquely one strain (strain specific) (Fig. 4). The pseudo-assembled genomes of the 6 strains contained distinct regions (Figs. 1 and 4). The left side of the draft genomes contained a large region carrying approximately 3700 PEGs that were in majority conserved at the genus (ca. 60% of PEGs) or at the species level (ca. 30% of PEGs). This high conservation of this large genomic region was expected as it corresponds to the scaffolds showing homologies to the chromosome of *E. meliloti* that carries most conserved housekeeping functions. A central variable part, ordered according to similarities with the symbiotic plasmid pSymA of *E. meliloti*, contained a higher proportion of strain specific genes or PEGs shared between strains from the same geographical origin which corroborated their respective associations to specific hosts that most probably rely on symbiotic plasmids (potentially shared locally) and very few genus or species conserved genes. The region showing similarities to the chromid pSymB of *E. meliloti* 1021 contained mostly species specific genes and to a lesser extent genus conserved genes (Fig. 4). Finally, the 3′ end of the draft genomes contained the remaining scaffolds which were not conserved at the species level and which included a high number of scaffolds in this region suggesting the presence of repeated sequences (Fig. 1). These latter scaffolds may be part of the most variable genomic fractions that is probably carried by accessory plasmid(s) identified in our strains. Furthermore, our results show that the Indian strain which present slightly larger genome sizes as compared to other *E. aridi* strains (Table 2) possess a larger proportion of genes that are specific to strains isolated from the same region (Fig. 4) suggesting acquisition of a higher number of accessory genes locally.

Based on assembly and scaffold ordering, recovery of genes involved in plasmid replication and mobilization (several copies of *rep*ABC, *trb* and *tra* genes) and on Eckhardt gel analysis, our results suggest that the genomes studied are composed by a chromosome of approximately 3.6 Mbp which included a majority of housekeeping genes that are conserved among *Ensifer* species, a chromid of 1.6–1.8 Mbp containing a majority of genes conserved at the species and to a lesser extent at the genus levels and large plasmid(s) which contained mainly accessory genes among which symbiotic *nod* and *nif* genes that most probably correspond to symbiotic plasmids. The genomic architecture and notably the presence of extra-chromosomal replicons representing nearly half the genomes that contains hyper variable genes including those involved in the symbiosis appears conserved among *Ensifer* and *Rhizobium* strains and species and differs from most *Mesorhizobium* or *Bradyrhizobium* species [49]. Despite the lack of complete genome sequences, our results further support such observation.

**Fig. 5:**
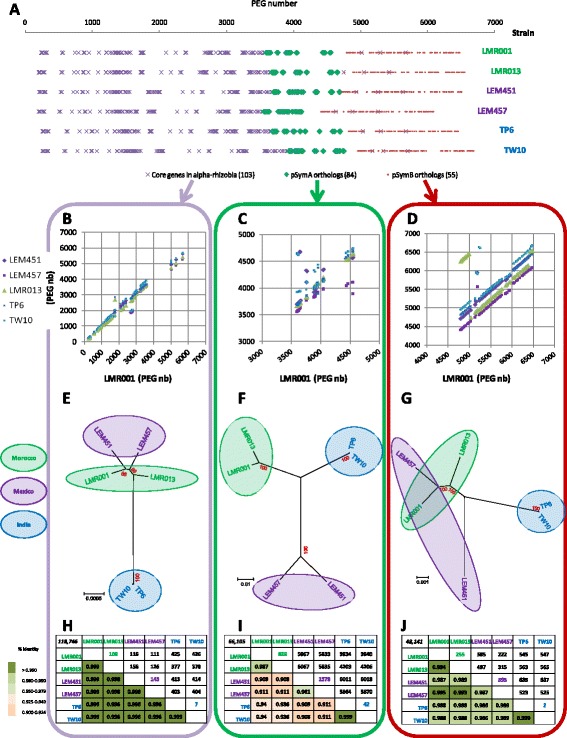
Synteny of predicted Protein Encoding Genes and evolutionary relationships among *E. aridi* strains in relation to PEG orthologies in *E. meliloti* 1021 replicons. PEG numbers corresponding to the 103 conserved genes among alpha-rhizobial strains and species as well as selected 84 and 55 orthologs from the 6 *E. aridi* strains (indicated at the *right* of the top plot) located in scaffolds showing synteny with, respectively pSymA and pSymB of *E. meliloti* 1021 are represented in (**a**). The synteny conservation between PEGs corresponding to these three gene sets (**b**, **c** and **d** respectively) were represented by plotting PEG numbers of the LMR001 strains against the other *E. aridi* strains (indicated on the *left side* of scatter plots). Evolutionary relationships of the corresponding gene sets upon concatenation (respectively **e**, **f** and **g**) were inferred using Neighbor Joining tree construction as described in Methods; and showed bootstrap values (from 1000 replications in *red*) near nodes, the scales at the *bottom* of each tree shows the number of substitutions per site. Strains originating from Morocco, Mexico and India were highlighted respectively in *green*, *purple* and *blue* (showed on the *left* of the trees). The matrix (**h**, **i** and **j**) show the respective number of variable sites (*top right matrix*) and percentage identities (*bottom left matrix*), the color code on the *left* of the matrix shows the % identity conservation obtained from pair-wise nucleotide sequence comparisons of the 3 concatenated and aligned gene sets obtained for the tree construction of **e**, **f** and **g** respectively. The number of positions aligned for each set is shown on the *top left* cell in each matrix

To explore the synteny level and the evolutionary relationships of genes conserved among the 6 desert strains, the 103 housekeeping genes used for the phylogenetic analysis and highly conserved among alpha-rhizobia, as well as genes that were mapped to the variable region showing homologies to *E. meliloti* 1021 pSymA (84 genes) or pSymB (55 selected genes) were retrieved, their order was compared and the overall evolutionary distances inferred upon concatenation and Neighbor Joining analyzes (Fig. 5). We found that the great majority of genes (100/103) identified as the most conserved among alpha-rhizobia were located on the putative chromosomes (Fig. 5a) and showed high synteny as estimated upon plotting of their respective gene order between the strain LMR001 and the other isolates (Fig. 5b). Genes present in the variable central region presented lower synteny confirming the high variability of this region that presents homologies to pSymA (Fig. 5c). In contrast, the 55 selected genes along the region showing homologies to pSymB showed high synteny despite a large rearrangement predicted in strain LMR013 (Fig. 5d). Intra-species evolutionary distances obtained when comparing 103 concatenated housekeeping genes principally located on the predicted chromosomes (Fig. 5e and h) indicated a close proximity between the Moroccan (LMR001 and LMR013) and the Mexican strains (LEM451 and LEM457) without clustering of the strains according to their geographical origin. These 4 strains that grouped together showed between 108 and 156 variable sites out of the 118,746 aligned nucleotides and were clearly separated (377–426 variable sites out of 118,746 aligned nucleotides) from Indian isolates (TP6 and TW10) that showed extremely high sequence conservation with only 7 variable sites out of the 118,746 aligned nucleotides (Fig. 5h). The intra-species evolutionary distances obtained when comparing genes presumably located on the symbiotic plasmids (Fig. 5f) showed a clustering of the strains according to their geographical origin with however a greater sequence divergence except for the comparison between the Indian isolates (Fig. 5f and i). The tree inferred from genes located in the region showing similarities to pSymB of *E. meliloti* 1021 indicated a greater divergence between the Mexican strain LEM451 and the strains LEM457, LMR001 and LMR013 (Fig. 5g and j) and again high sequence conservation between the two Indian strains TP6 and TW10. The % identity between the concatenated gene sets varied from 0.996 to 0.999 when the 103 genes conserved among alpha-rhizobia were compared, from 0.986 to 0.999 when the 55 concatenated gene sequences located on scaffolds showing similarities with the chromid pSymB of *E. meliloti* were used and from 0.908 to 0.999 for the 84 concatenated gene sequences located in the pSymA syntenic region (Fig. 5h, i and j). Overall, these results showed that the two Indian strains share a more conserved genome with regard to other pair-wise comparisons which corroborates ANI and *in silico* DDH results (Fig. 2). The phylogenies of the concatenated gene sets located in regions showing homologies to the chromosome and the chromid of *E. meliloti* did not cluster the Moroccan and the Mexican strains according to their geographical origin indicating a closer evolutionary relationship.

The proportion and composition of the core genome in the new species was compared to those of 3 *Ensifer* species for which more than 6 genome sequences were available at the time of writing (ie. *E. meliloti*, *E. medicae* and *E. fredii*). Using the method developed by Ozer and colleagues [37], the sizes of core and accessory genomes as well as the GC % of each fraction were estimated and the PEGs assigned to each fraction (predicted upon RAST annotation) were retrieved (Table 2 and Additional file 2: Figure S4). The size of the core genomes varied between species. The smallest core genome was that of *E. aridi* with 5.12 Mbp, *E. fredii* and *E. meliloti* indicated respectively core genomes of 5.55 and 5.76 Mbp and the largest estimated core genome was found for *E. medicae* with 5.95 Mbp. Core genomes represented from 75 to 87% of the genomes (Additional file 2: Figure S4). The GC % of accessory genomes were lower (58.8–60.1%) than those corresponding to the core genomes (61.4–62.8%) for all 4 species studied. The GC profile and segmentation points along pseudo-assembled genomes were investigated in the 6 *Ensifer aridi* strains using GC-Profile [53] and indicates that the region corresponding to the scaffolds in synteny with *E. meliloti* pSymA presented a lower and more variable GC % (data not shown) which based on our analyzes contain most of the accessory genomic fraction in our desert species. On the basis of SPINE analysis it was also observed that the average (range) of accessory genome size in *E. aridi* was comparatively larger (1576 Kbp {1324–1737}) than those of *E. meliloti*, *E. medicae* and *E. fredii*. Interestingly, the GC profiles obtained from pseudo-assembled genomes and the size of the three regions in our genomes are in line with the description made earlier regarding the replicon (chromosome, chromid and plasmid) general characteristics [54].

Taken together, the facts that this variable region (Figs. 1 and 4) shows evolutionary relationships that correlate with host and geographical origin (Fig. 5f) and contains symbiosis specific, plasmid replication and mobilization genes and the presence of large plasmids as revealed by Eckhardt gel analysis demonstrates the presence of such replicons in this species which is common among *Ensifer* species [49]. Furthermore, given the evolutionary distances obtained on the three putative replicons and their corresponding conservation levels (genus, species, strain) (Fig. 5), our data support the evolutionary scenario presented by Gallardini and colleagues on the differential evolutionary routes multipartite genomes may undergo as exemplified in *E. meliloti* [55]. Nevertheless, additional experiments are required to further test this hypothesis and notably estimate the relative evolutionary routes undertaken among isolates belonging to this new species.

Based on 6 genomes per species and using sequence identities of 80% on 80% of sequence alignments as cutoff values, the pan-genomes of *E. aridi*, *E. meliloti*, *E. medicae* and *E. fredii* included 10,768, 10,326, 9622 and 10,624 putative gene clusters contained within 9.36, 9.12, 8.68 and 9.60 Mbp respectively. The tBlastX results using the aforementioned cutoffs for the 6 strains included in the analysis and for each species on their respective pan-genomes are represented in Additional file 2: Figure S5. The large pan-genomes identified in the 4 species support the wide phenotypic versatility among *Ensifer* species and strains that have to adapt to various environments. There were however differences between the species with a greater genomic diversity found in *E. fredii* and *E. aridi* which may be related to the broader diversity of hosts with which they may be associated. Despite a similar proportion of core and accessory genomes in strains belonging to these two species (Additional file 2: Figure S4A), the size of the core genome and the pan-genome analysis indicated a lower proportion of conserved protein shared by all *E. aridi* strains. However, this conservation level was higher when strains from the same geographical origin were compared. The larger diversity of proteins in accessory genomes in this species and the greater conservation between strains isolated from the same region further support the presence of variable large plasmid(s) acquired locally.

### Functional genomics

**Fig. 6:**
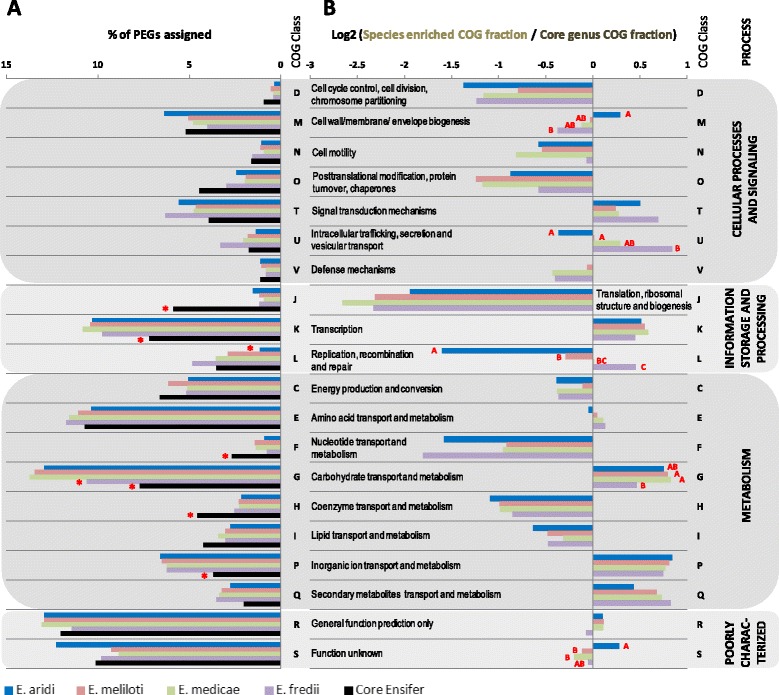
COG classification of *Ensifer* species specific and genus conserved gene sets. The *left side* horizontal histogram (**a**) shows the percentage of predicted PEGs from conserved gene sets as classified in COG classes and processes. The COG classification of the core *Ensifer* proteome at the genus level (estimated using BBH with 70% identity threshold from 28 predicted proteomes from 8 species) is shown as *black bars* on the left side graph. Predicted proteomes conserved in *E. aridi* (*blue*), *E. meliloti* (*pink*), *E. medicae* (*green*) and *E. fredii* (*purple*) were obtained by subtracting the core *Ensifer* proteomes to core species proteomes (See Methods). The right side horizontal histogram (**b**) shows for each COG class the logarithmic base 2 ratios of the species conserved gene set fraction by that found in the core *Ensifer* gene set. A negative value indicates higher representation of the corresponding class in the core genus gene set as compared to the species conserved gene set and conversely, a positive value suggests that the corresponding class is more represented in the species conserved gene set. The color code used for the species enriched or core *Ensifer* gene sets is indicated at the *bottom* of the graph. COG classes, their respective descriptions and the process to which they belong to are indicated in *black text* in the figure. Values that are significantly different (*p* value < 0.05) within a COG class upon comparison of k proportions using the Marascuillo procedure as implemented in XLStat are indicated by a *red* “*” (**a**) and different *red letters* in (**b**) indicate intra-class significant differences in the log2 ratios between species conserved and genus conserved gene set fractions

To explore the functional attributes associated to the new desert rhizobia and compare them to other *Ensifer* species, we used the COG and KEGG classifications of conserved gene sets that were predicted in each of the 4 species *Ensifer meliloti*, *E. medicae*, *E. fredii* and *E. aridi*. The number of PEGs identified in each core species genome and results from COG assignments are reported in Table 2. We found that the core genome of the new *E. aridi* species contains a smaller number of PEGs when compared to the other *Ensifer* species. This difference may be due to the relatively lower (i) genome size (Table 2) and (ii) proportion of the core genome (Additional file 2: Figure S4) found in this new species. To further compare the functions associated to the new desert species, we then subtracted from the core gene sets (core genomes at the species level), those that were conserved at the genus level and which included 2539 putative genes. The number of PEGs corresponding to species conserved gene sets (species enriched), those corresponding to the core *Ensifer* genus and the proportions of PEG with COG assignment are presented in Additional file 2: Figure S6. If the number of genes identified as species conserved in the new species was slightly lower as compared to the other *Ensifer* species, the proportion of genes with assigned COG class(es) was globally conserved across species (ranged from 64.7 to 68.2%) and the genes that were identified as conserved at the genus level showed more than 92.4% of COG assignments (Additional file 2: Figure S6). Being conserved across species and strains, this higher functional classification was expected as these genes have more chances to be essential and include housekeeping functions that have been more thoroughly studied. The relative abundance of each COG class was estimated for the 4 compared core *Ensifer* genomes (Fig. 6a). To highlight variations in the COG classification between core proteomes at the genus level (Corresponding to essential conserved functions in the *Ensifer* genus) and the species conserved fractions, for each functional class, we calculated and log2 transformed ratio of the Species enriched associated COG fraction by the “Core genus genome” associated COG fraction, and we represented the results as horizontal histogram (Fig. 6b). We found that, regardless of the species compared, COG classes D, O, J, C, N, F, H and I were overrepresented in the conserved genus fraction while classes T, K, G, P and Q were inversely more represented in the “species conserved” fractions. Classes J (Translation, ribosomal structure and biogenesis), F and H (involved in the transport and metabolism of nucleotides and coenzyme respectively) were significantly more represented in the genus conserved fraction. As expected, the most significant difference between genus and species conserved fractions was found for the class J which include all components of the translational machinery that is particularly conserved at the interspecies level. Conversely, classes K (Transcription), G and P (involved in the transport and metabolism of carbohydrates and inorganic ions respectively) were significantly more represented in the 4 species compared suggesting diversification of associated functions in the genus. A recent study on the model *E. meliloti* showed that upon curation of pSymB (chromid) and pSymA, the resulting variant consisting of a chromosome to which pSymB essential genes were integrated showed a marked reduction in carbon sources it could utilize with only 19 C source instead of the 74 C that wild type strain can take up and metabolize [56]. Additionally, several nitrogen, phosphorus and sulfur sources were impacted by the lack of plasmid and chromid replicons. The study by diCenzo and colleagues shows that dispensable replicons, which contain mostly accessory genes, enable the bacterium to widen its niche range. Our results supports such observation as COG classes G and P are over-represented in the species enriched fractions as compared to the genus conserved fraction.

Despite a similar proportion of PEGs with COG assignment in the 4 compared conserved species proteomes (Additional file 2: Figure S6), we found notable differences in the relative proportions of COG classes between species (Fig. 6). In *E. aridi*, we found a higher proportion of PEGs assigned to the classes M (Cell wall/membrane/envelope biogenesis) and S (functions unknown) in the species conserved fraction relative to the genus conserved fraction. In contrast, classes U (Intracellular trafficking, secretion and vesicular transport) and L (Replication, recombination and repair) showed the opposite (Fig. 6b). The higher proportion of genes in class M indicates that *E. aridi* has favored, in contrast to the compared species this cellular process that may be essential for sustainability in deserts. Interestingly, *E. aridi* also showed a higher proportion of poorly characterized class S COGs that have unknown functions which could also participate in sustainment in desert. The lower proportion of species conserved genes categorized in class L in *E. aridi* was significant as compared to other species and was somehow surprising as one could expect a higher number of genes associated to replication, recombination and repair functions as shown for example for *Deinococcus deserti* [57]. This observation suggests that other mechanisms may participate in the tolerance to abiotic stresses in this new *Ensifer* species. Furthermore, the lower amount of items identified in the new species core genome COG class L that also include mobile proteins and other phage related elements may also suggest that it is less subjected to horizontal gene transfer (HGT) and genomic rearrangement as compared to the 3 other rhizobial species despite a higher proportion of accessory genes that may result however from a greater variability of plasmids acquired.

The number of phage and virus related sequences as well as insertion sequences that participate to intra-genomic recombinations were estimated using PHAST and ISsaga pipelines respectively in the 4 compared species. The presence of IS and prophage was predicted in all strains and species which support the capacity of symbiotic nitrogen fixing bacteria to acquire genes from related or more distant species, and to concomitantly undergo intra-genomic rearrangements that may partly explain their “mosaic” type of genomic architecture and their capacity to adapt to contrasted environments (from intracellular to saprophytic life styles) [36, 55, 58–60]. However, the frequency of prophages and IS varied both at the intra- and inter-specific levels (Additional file 2: Figure S7). Regarding prophage sequences, for each species, intact prophage sequences were predicted in at least 2 strains out of 6 analyzed here. The lowest numbers of predicted phage related regions were found in *E. aridi* and *E. fredii* while *E. medicae* presented the greatest number of intact or questionable prophage sequences. The number of IS also showed marked differences between strains and species (Additional file 2: Figure S7). Considering all IS predicted by ISsaga, the mean number of IS per genome were respectively 78.5, 131.5, 212.3 and 180.3 for *E. aridi*, *E. meliloti, E. medicae* and *E. fredii*. These IS were classified in 39 IS families and their respective frequencies are reported in Additional file 1 (Table S6). The mean numbers of IS families predicted in *E. aridi*, *E. meliloti, E. medicae* and *E. fredii* were respectively 17.1, 19.7, 22 and 29.5 which shows great differences between species. Five IS families were predicted in all strains and species (IS66, IS3_ssgr_IS407, IS21, ISNCY_ssgr_IS1202 and ISAs1). Globally, we found a relatively low IS diversity and density in *E. aridi*, a higher IS density in *E. medicae* and a higher diversity of IS families in *E. fredii* as compared to other species. The IS density among *Ensifer* species was highly correlated to the number of items identified in COG class L observed from functional genomics analyzes and explain the variability observed in this particular class (data not shown). These results suggest that in comparison to other species studied here, *E. aridi* strains possess a lower diversifying agent potential which could limit their adaptation to other ecological niches [61, 62]. However, we found in the genomes of *E. aridi* that predicted prophage regions were mainly incomplete and intact prophage regions were only identified in the Indian strains suggesting a more recent transfer in Asian isolates. The higher IS density and intact prophage regions predicted in the Indian isolates suggest a higher potential of recombination in these strains as compared to Moroccan and Mexican ones that may result from a more recent transfer event.

With the objective to identify functions associated to the new *Ensifer* species, RAST predicted protein sequences from proteomes conserved by each one of the 4 *Ensifer* species compared were further subjected to pathway reconstruction using KEGG tools which enables to retrieve functional modules (regrouping several KO entries). We found that only half of the predicted proteins conserved at the species level had a KO assigned in the 4 species (respectively for *E. aridi*, *E. meliloti*, *E. medicae* and *E. fredii*: 50.2, 49.9, 47.3 and 50.3%; data not shown) which shows that many functions remain to be identified in this genus. Nevertheless, based on current knowledge of orthologous protein functions and interplay in a defined pathway or structural complex (functional modules), it remains possible to compare proteomes *in silico* and identify the presence of currently known functions. Using the module reconstruction tool on the core species proteomes from which the conserved genus proteome was subtracted, we could rapidly identify functions associated to a species. The Additional file 1 (Table S7) shows the results obtained upon module reconstruction in the 4 *Ensifer* species compared. Using this comparative tool, we found 8 modules that appear species conserved (predicted in the 6 strains studied) while not identified in the conserved genes set shared by the 8 *Ensifer* species. Surprisingly, 6 out of 8 of these species conserved modules were predicted in *Ensifer fredii*. Some of these functions may participate in the broad host range found in this species as exemplified by strains HH103 and USDA257 [5, 63]. The remaining two species conserved modules were found in *E. aridi*. These were two ATP binding cassette (ABC) type transport systems for octopine/nopaline (M00231) and inositol phosphate (M00599) that both rely on 4 units (a substrate binding protein, 2 permease proteins and an ATP binding protein). According to KEGG based orthologies and specific blast searches, the module M00231 (involved in octopine/nopaline transport system) was predicted in 2 copies for both the Indian and the Moroccan strains while a single copy was found in the both Mexican strains. Being identified from the core genome of *E. aridi*, this transport system is also present in other root nodulating bacterial strains or species but its frequency may vary at the intra-species level (data not shown). Given the phylogenetic proximity between symbiotic rhizobia and *Agrobacterium* from which these genes whose function were described, and the sharing of their habitats, this transport system has possibly evolved from ancient HGT events which may provide improved fitness to the bacterial strains capable of utilizing such compounds [64]. Furthermore, a possible affinity of this transport system toward amino acids or derivatives may have enabled to broaden/ameliorate the nutrient uptake capacity in the new *Ensifer* species presented here. The second system that was predicted in all 6 sequenced strains of *E. aridi* was revealed by the prediction of the complete KEGG module M00599 that refers to an ABC type inositol phosphate transport system. The scarcity of such module among rhizobia as referred to KEGG orthology distribution indicates that it may be seen as a particular trait associated to the presently described species. Inositol phosphates and derivatives constitute the majority of soil organic phosphates and the presence of this transport system may be essential for survival in a nutrient poor environment such as desert. This capacity may enable the bacterium to utilize phosphorylated inositol derived compounds originating from the plant degradation which would further improve the bacterial fitness and participate in its survival by providing utilizable carbon and phosphorus sources upon plant/nodule senescence. Furthermore, if a myo-inositol phosphate synthase was predicted in all *E. aridi* strains, this transport system may also, together with derived compounds, participate in the osmotolerance of the symbionts [65] and contribute to the particular adaptation of this new rhizobium toward environmental stresses such as those found in desert biome. Attempts to grow selected strains using octopine or nopaline as sole carbon sources in AT medium produced negative results, however, we could not conclude that these compounds are not transported but that under these conditions, such C sources were not transported and/or utilized by these bacteria. Indeed, additional biochemical and genetic studies are required to identify the substrates’ affinities of the ABC type transporters identified here *in silico* and characterize their functional roles which are outside the scope of this report. Nevertheless, to improve our knowledge on this new species, several phenotypic characters were studied including (i) the potential utilization of various carbon sources, (ii) their tolerance to salt, pH, temperature and antibiotics and (iii) their capacity to nodulate several legumes of potential interest.

### Phenotypic characterization of *Ensifer aridi*

To study the phenotypic characteristics of the new taxon identified here, the sequenced strains were screened for their capacity to utilize a wide range of carbon sources using the Biolog GN2 microplate system and compared to those of *E. meliloti* 1021. The results of the phenotypic macro-arrays are presented in the Additional file 3. We found that the great majority of carbohydrates screened could be utilized by all *E. aridi* strains except xylitol. The wide range of carbohydrates rhizobia can utilize is expected as these microorganisms are mainly growing in soils which requires the capacity to utilize a broad range of carbohydrates for their nutrition. Even though the relative capacity varied with the chemical and the strain, all *E. aridi* strains were also able to utilize Pyruvic acid methyl ester and Succinic acid methyl esters, the following carboxylic acids: Acetic acid, Formic acid, D-Glucosaminic acid, b-Hydroxybutyric acid, D,L–Lactic acid, Malonic acid, Propionic acid and Succinic acid; Glycerol and the amino acids and derivatives : L-Alanyl-glycine, L-Asparagine, L-Aspartic acid, L-Glutamic acid, L-Histidine, Hydroxy-L-Proline, L-Ornithine, L-Proline, D,L-Carnitine, γ-Aminobutyric acid; the aromatic chemical Uridine and finally the brominated compound Bromosuccinic acid (Additional file 3). It should be noted that a selection of 16 carbohydrates tested in GN2 plate were also tested using the HiMedia disc fermentation assays which indicated contrasting results and demonstrate that the basal medium used can affect metabolic capacities in the studied strains. Interestingly, among compounds that were utilized by all desert strains, the malonic acid was efficiently metabolized and the only substrate that was not used by *E. meliloti*. Previous phenotypic tests on various rhizobia including *Ensifer* strains belonging to *E. meliloti*, *E. fredii*, *E. saheli* and *E. terangae* [66] supports the originality of such metabolic capacity among *Ensifer* species which is however identified in *R. leguminosarum* as shown by a recent report showing that the majority of the 72 *Rhizobium leguminosarum* strains screened phenotypically were capable of using this carbon source [67]. Malonate utilization most probably rely in the presence of the *mat*RABC locus shown to be involved in the regulation through a GntR type transcriptional regulator encoded by *mat*R [68], a malonyl-coenzyme A (CoA) decarboxylase encoded by *mat*A, a malonyl-CoA synthetase encoded by *mat*B and a probable malonate carrier protein encoded by *mat*C in this species [69, 70]. However, our results on *E. meliloti* 1021 differ from a previous study which demonstrated that this strain was capable of growth in media containing this organic acid as sole Carbon source which was genetically linked to *mat*PQMAB genes and relied on a tripartite ATP-independent permease (TRAP) system for import encoded by the three genes *mat*PQM [71]. Nevertheless, in the latter study, the growth was very slow when compared to glucose containing media and no growth was observed during the first 3 days which could explain the difference with our report since we only recorded results after 3 days. The use of malonate as a carbon source was further tested for three strains of *Ensifer aridi* (LMR001, LEM451 and TP6) and *E. meliloti* 1021 upon a 5 days growth in the minimal growth medium AT complemented with malonate (40 mM), succinate (40 mM) or a mixture of succinate and malonate (20 mM each). The results shown in Additional file 2: Figure S8 confirmed the ability of all the strains tested to grow on malonate as the sole carbon source but this growth was very limited for most of the strains. When compared to the medium containing succinate as sole carbon source, the growth was nearly identical for the Indian strain TP6 which contrasts with the Mexican strain LEM451 (34%), the Moroccan strain LMR001 (23%) and *E. meliloti* (9%). More interestingly, using a mixture of the both carbon sources but at a concentration of 20 mM each, we found that, compared to the succinate, the growth was significantly increased in the three *E. aridi* strains tested while it was reduced by a third in the reference *E. meliloti* strain. This result demonstrates the efficiency to utilize malonate in desert *Ensifer* strains and suggests the presence of functional import system(s) and metabolism for this carbon source despite the competitive effect it has on succinate dehydrogenase which leads to a reduced tricarboxylic acid (TCA) cycle functioning [72]. The genomes of the desert *E. aridi* strains were screened for the presence of genes involved in malonate uptake and metabolism which led to the discovery of a locus containing the *mat*RABC genes that were in synteny with those of *R. leguminosarum*. Specific Blast searches were performed on genomes from the *Sinorhizobium/Ensifer* clade and showed that it was also present in some *E. fredii* strains as well as in the sequenced *E. saheli* strain USDA4893 which was however showing no growth in a previous report [66] and which suggests the involvement of additional genes for efficient malonate anabolism. The high level of malonate found in many leguminous plants including roots and nodules and studies on *mat*B mutant suggested that this C_3_-dicarboxylic acid could be involved in symbiotic nitrogen fixation (for review, see [70]). However latter studies on *E. meliloti* [71] and *R. leguminosarum* [73] *mat* mutants showed that malonate is not a driver of the biological N_2_ fixation and genes involved in its metabolism are dispensable for proper symbiotic function although this may depend on the malonate level in nodules which varies with the plant species and/or growth conditions. Interestingly, given the high proportion of the potentially toxic organic acid malonate in roots and nodules found in some leguminous plants, the presence of mechanisms that can import and metabolize efficiently this compound such as that found in *R. leguminosarum* and few other species may be seen as a competitive advantage towards other root colonizing microorganisms including symbiotic ones and pathogens. In the case of the desert rhizobial strains, such metabolic capacity may be required so as to optimize infections and successful nodulation when the microorganism meets host roots which are rare in desert soils.

The studied strains were also tested for their growth at high temperatures and on media that contained bromothymol blue, various pH, NaCl concentrations or antibiotics (Additional file 1: Table S8). The results suggest that like other *Ensifer* species, *E. aridi* strains are acid producing, fast growing and tolerate high NaCl concentrations. Strain TP6 showed however a lower tolerance toward high salt concentrations as it didn’t show growth when 2% of NaCl was added to the culture medium. Furthermore this latter strain also presented a particularly high antibiotic resistance spectrum as it was growing in presence of Chloramphenicol, Kanamycin, Streptomycin, Nalidixic acid and Spectinomycin while the strains from Morocco and Mexico presented tolerance to only Nalidixic acid and Spectinomycin. If the strain TW10 also resisted to Nalidixic acid, it also resisted to Kanamycin but was sensitive to Spectinomycin. We found that all strains were capable of growth at 48 °C which has not been reported in other defined *Ensifer* species and at high pH which would be expected as the soils sampled were all alkaline (ranging from 7.8 to 9.2) [10, 11], (Rocha et al., unpublished data) and temperatures in the sampled soils may often reach such extremes.

*Ensifer aridi* strains isolated from different continents were tested for their nodulation ability on selected legume species (crops and wild/native) belonging to all the three subfamilies of Fabaceae (Additional file 1: Table S1). These strains are effectively nodulating various arid leguminous trees which can be used for arid reforestation programs. The difference in the host range between different strains can be explained on the basis of the individual symbiosis related gene phylogenies [10, 11], (Rocha et al., unpublished data) as well as through the comparative analysis of the accessory genome sequences that are, according to the data presented in this report, probably carried by large plasmids. Although the Mexican *E. aridi* strain isolated from *Phaseolus filiformis* nodulated several species including *Vachellia*, *Senegalia* and *Prosopis*, it did not form nodules on *V. gummifera* and species of *Tephrosia* from which the other strains were isolated. All the *E. aridi* strains failed to develop nodules with *Mimosa*, *Glycine max* and *Arachis hypogaea* which suggests that the nodulation of these tested legumes require more specific symbiotic determinants.

## Conclusions

Using comparative genomics and current genome based species delineation tools, a new *Ensifer* species that appears particularly adapted to hot desert could be characterized. Despite a majority of genes with no function assignments, the KEGG based analysis performed on the species specific proteome enabled to identify two ABC type transporters possibly involved in the transport of inositol phosphate and octopine/nopaline that remain however to be functionally characterized. Furthermore, the phenotypic macroarrays enabled the identification of malonate as a carbon source it can utilize efficiently and which may have broaden its fitness toward legumes that produce this organic acid. Such conserved functions may participate in its survival in desert biome where these strains were recovered and may be further studied. A large accessory gene set that is probably carried by plasmids that varied in size among isolates may in part explain the different plant species they are able to nodulate. Being particularly adapted to high temperatures, aridity and alkaline type of soils, being present on three continents, *E. aridi* appears to have acquired locally divergent symbiotic gene sets. Given these particular traits, this new species deserves interest notably for its potential in biotechnology. Its capacity to host variable symbiotic determinants suggest competency in transferring/acquiring symbiotic determinants through HGT such as plasmid transfers that may enlarge its host range and our results indicated the loss of symbiotic genes from one of the strain that most probably resulted from plasmid curing following subculturing. Looking to symbiotaxonomy of *E. aridi* strains, it is suggested that they are promiscuous and mainly nodulate members of mimosoideae and papilionoideae including annual herbaceous crop legumes (species of *Vigna*, *Cyamopsis tetragonoloba*) and perennial trees (*Acacia* and *Prosopis*). Cross nodulation studies and the inter and intra host ranges described here is interesting and further supports its acquisition of *nod*-*nif* kits from local rhizobia associated with host in that particular ecological niche. During the long term host-rhizobia co-evolution, soil factors and the host selection pressure resulted in diversification of symbiotic properties of these rhizobia. Finally, the distribution of this new species in desert biome in three continents raises several questions which remain to be investigated. Notably, the reason why they are until now only recovered from desert type of biome at such distant geographical locations. Are these bacteria part of the microbial cortege which are transported through desert sand storms and which have been shown to enable microbial trans oceanic trips [74–79]. If such hypothesis could explain the presence of this species in the three continents, the reason regarding its recovery from arid environments is however puzzling. Indeed, one would expect a wider spread through transported sands that are deposited over large regions and which should have enabled its recovery from more types of biomes. A poor competitivity when in presence of indigenous rhizobia from other types of environments which favor microbial densities could partly explain this geographical repartition. In line with this assumption, the low IS density found in this new species suggests that it is less armed in tools that generate the diversity required to adapt to environmental changes that should however be balanced through large accessory gene sets acquisition capacities. Further studies aiming at clarifying the structure of their genomes and their evolution will be needed to address such questions.

## Additional files

Additional file 1: Table S1. Nodulation tests on selected leguminous plants. **Table S2.** Primer efficiencies and correlation factors obtained from quantitative PCR assays. **Table S3.** List of strains and species used for the phylogenetic analysis (Table S3A) and list of 103 *E. meliloti* 1021 genes used for the phylogenetic analysis (Table S3B). **Table S4.** PEG lists from desert isolates corresponding to the 103 most conserved genes among alpha-rhizobia (Table S4A), 84 orthologous genes carried by pSymA in *E. meliloti* 1021 (Table S4B) or 55 selected genes carried by pSymB (Table S4C). **Table S5.** Genome sequencing and assembly overviews. **Table S6.** Frequency of predicted ISs in relation to IS families in the 4 compared *Ensifer* species. **Table S7.** Identification of species specific and complete KEGG modules in the 4 compared *Ensifer* species. **Table S8.** Phenotypic characteristics. (XLSX 63 kb)
Additional file 2: Figure S1. Eckhardt gel electrophoresis showing replicon size and number in the newly sequenced *Ensifer* strains. **Figure S2.** Quantification of the copy number of chromosomal and symbiotic markers from gDNA extracts of strains LEM451 and LEM457 cultivated in different media. **Figure S3.** Horizontal box plot showing the distribution of the % identities of 1 Kbp fragments used for pair-wise ANI calculations. **Figure S4.** Comparison of core and accessory genomes proportion, size and GC content between compared *Ensifer* species. **Figure S5.** Pan-genome size in the 4 compared *Ensifer* species (based on 6 genomes per species). **Figure S6.** COG classification of *Ensifer* species- and genus- conserved PEG sets. **Figure S7.** Predicted insertion sequences and prophage regions in the 4 compared *Ensifer* species. **Figure S8.** Malonate utilization assay. (PPTX 787 kb)
Additional file 3: Ability of the new species to metabolize carbon sources in Biolog GN2 microplates compared to references strains *E. meliloti* 1021. For each strain, the data represent the mean of two replicates and is expressed as the % to maximum activity. The color code strengthen the relative activity of each carbon source ranging from no activity (*white* when <20% of maximal activity), and 4 levels of grey which correspond to ranges from weak activity (*light grey*) to high activities (*dark grey*) using respectively intervals ≤40, ≤60, ≤80 and ≤100%. Carbohydrates fermentation using the HiMedia disc assay are indicated (*) and *red numbers* indicate no fermentation activity using this method. (PPTX 121 kb)

## Abbreviations

ABC: ATP binding cassette
ANI: Average nucleotide identity
BBH: Bidirectional best hit
COG: Clusters of Orthologous Groups of proteins
Ct: Cycle threshold
DDH: DNA-DNA Hybridization
gDNA: genomic DNA
GFP: Green fluorescent protein
GGDC: Genome to genome distance Calculator
HGT: Horizontal gene transfer
IS: Insertion sequence
Kbp: Kilo base pairs
KEGG: Kyoto Encyclopedia of genes and genomes
KO: KEGG Orthology
Mbp: Million base pairs
MLSA: Multi locus sequence analyzes
NGS: Next generation sequencing
NJ: Neighbor joining
PEG: Protein encoding gene
RDM: Rhizobia defined medium
RMS: Rhizobium minimal medium with Succinate as carbon source
RNB: Root nodulating bacteria
RPS: Reverse position specific
SAV: Sequence analysis viewer
SD: Standard deviation
TY: Tryptone yeast extract
WGS: Whole genome shotgun
YEM: Yeast extract mannitol
