# Supplementary material for: Genomic characterization of Ensifer aridi, a proposed new species of nitrogen-fixing rhizobium recovered from Asian, African and American deserts
Source: BMC Genomics. 2017 Jan 14;18:85. doi: 10.1186/s12864-016-3447-y (PMC5237526; doi:10.1186/s12864-016-3447-y)
Supplement: Additional file 2: Figure S1. — Eckhardt gel electrophoresis showing replicon size and number in the newly sequenced Ensifer strains. Figure S2. Quantification of the copy number of chromosomal and symbiotic markers from gDNA extracts of strains LEM451 and LEM457 cultivated in different media. Figure S3. Horizontal box plot showing the distribution of the % identities of 1 Kbp fragments used for pair-wise ANI calculations. Figure S4. Comparison of core and accessory genomes proportion, size and GC content between compared Ensifer species. Figure S5. Pan-genome size in the 4 compared Ensifer species (based on 6 genomes per species). Figure S6. COG classification of Ensifer species- and genus- conserved PEG sets. Figure S7. Predicted insertion sequences and prophage regions in the 4 compared Ensifer species. Figure S8. Malonate utilization assay. (PPTX 787 kb) [file 12864_2016_3447_MOESM2_ESM.pptx]

## Slide 1
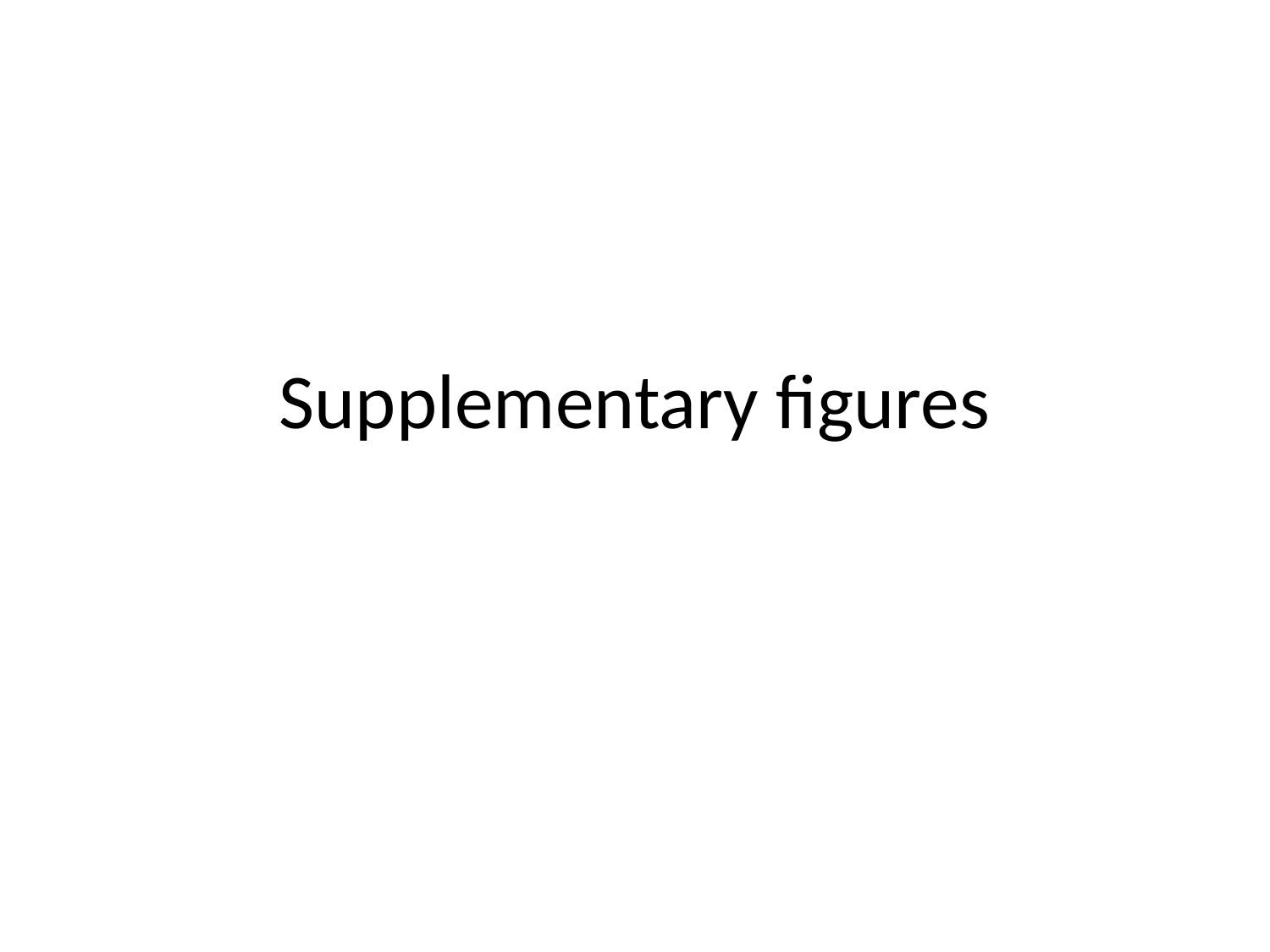

# Supplementary figures

## Slide 2
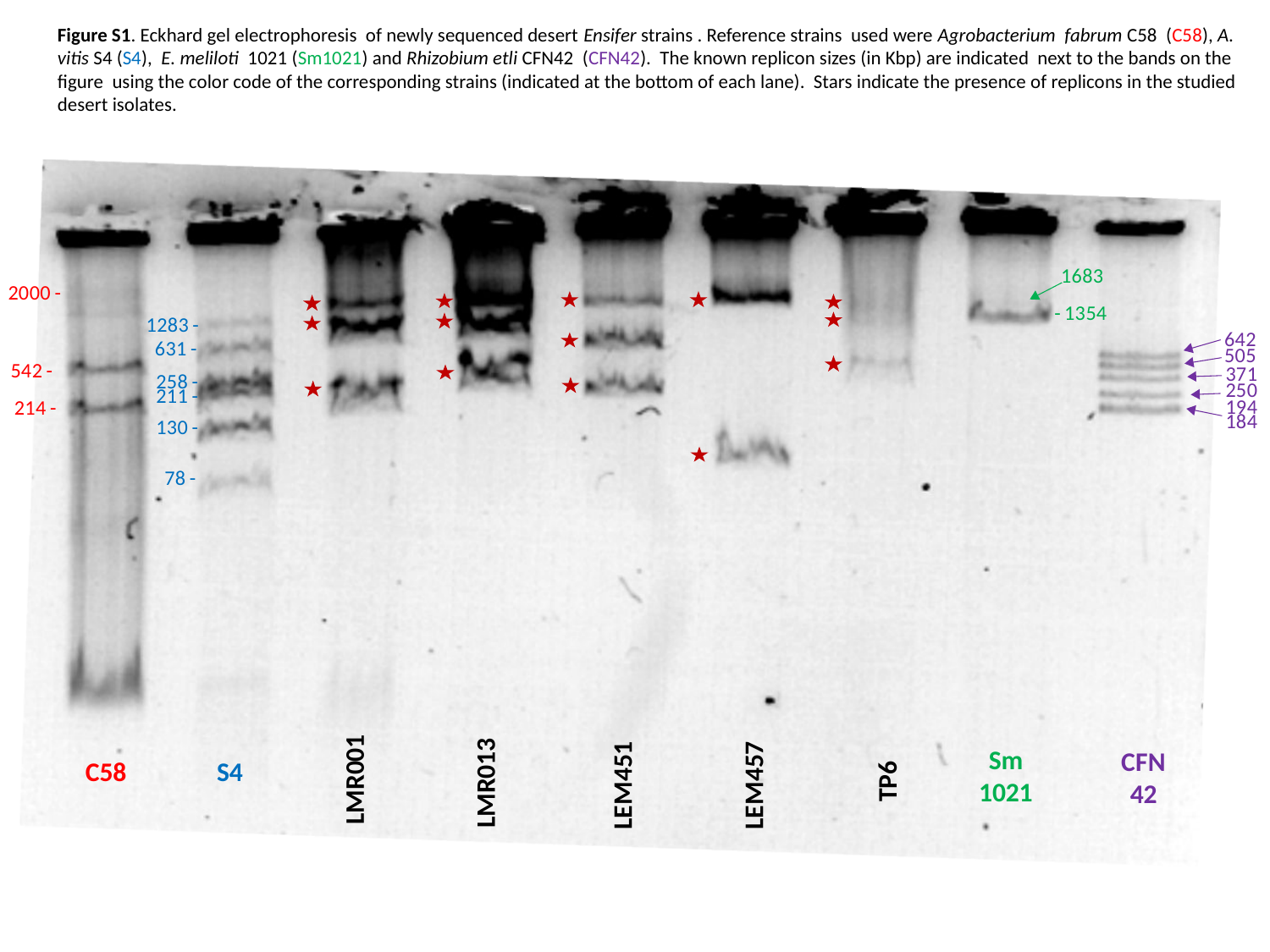

Figure S1. Eckhard gel electrophoresis of newly sequenced desert Ensifer strains . Reference strains used were Agrobacterium fabrum C58 (C58), A. vitis S4 (S4), E. meliloti 1021 (Sm1021) and Rhizobium etli CFN42 (CFN42). The known replicon sizes (in Kbp) are indicated next to the bands on the figure using the color code of the corresponding strains (indicated at the bottom of each lane). Stars indicate the presence of replicons in the studied desert isolates.

## Slide 3
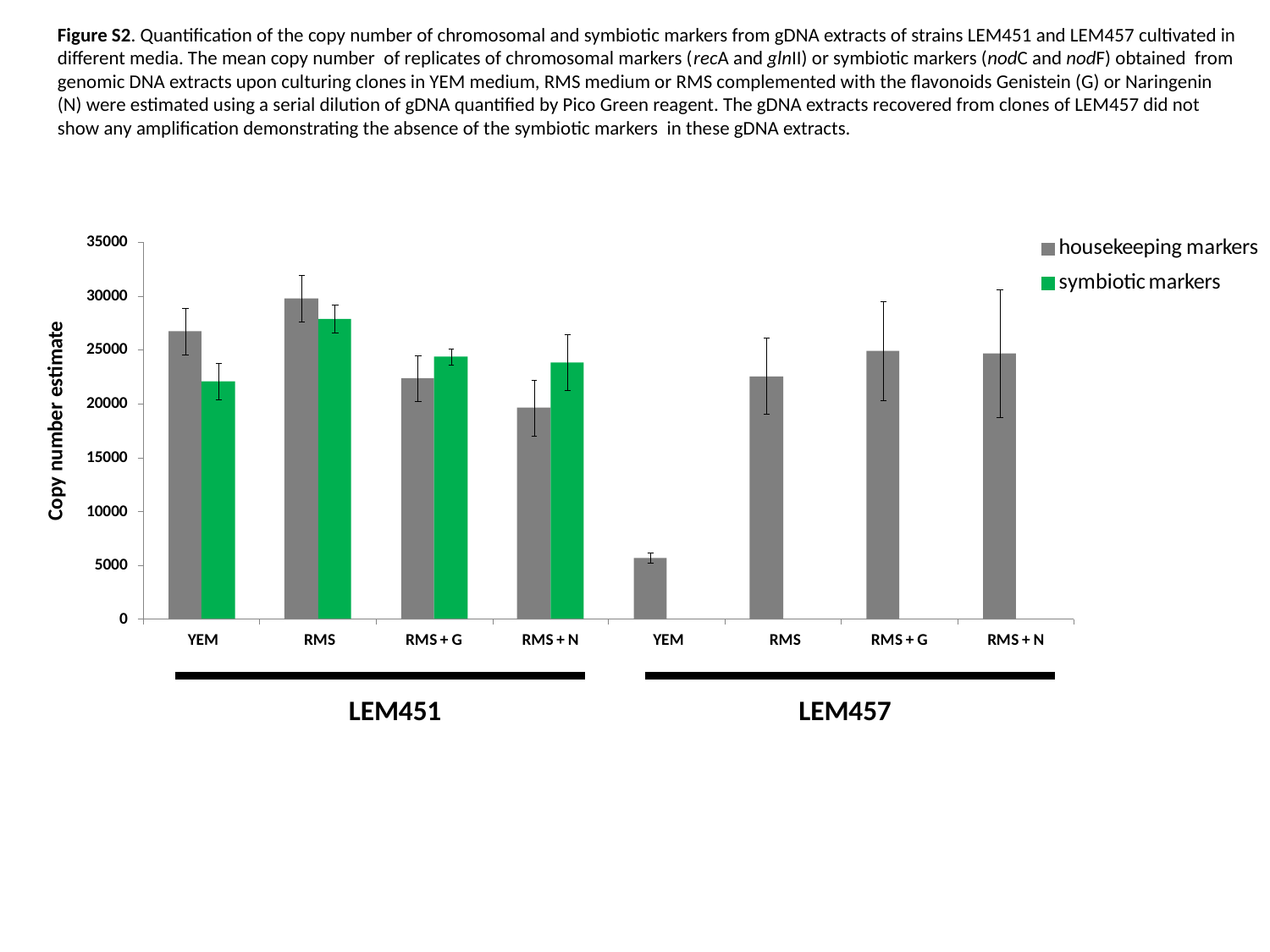

Figure S2. Quantification of the copy number of chromosomal and symbiotic markers from gDNA extracts of strains LEM451 and LEM457 cultivated in different media. The mean copy number of replicates of chromosomal markers (recA and glnII) or symbiotic markers (nodC and nodF) obtained from genomic DNA extracts upon culturing clones in YEM medium, RMS medium or RMS complemented with the flavonoids Genistein (G) or Naringenin (N) were estimated using a serial dilution of gDNA quantified by Pico Green reagent. The gDNA extracts recovered from clones of LEM457 did not show any amplification demonstrating the absence of the symbiotic markers in these gDNA extracts.
Copy number estimate
LEM451
LEM457

## Slide 4
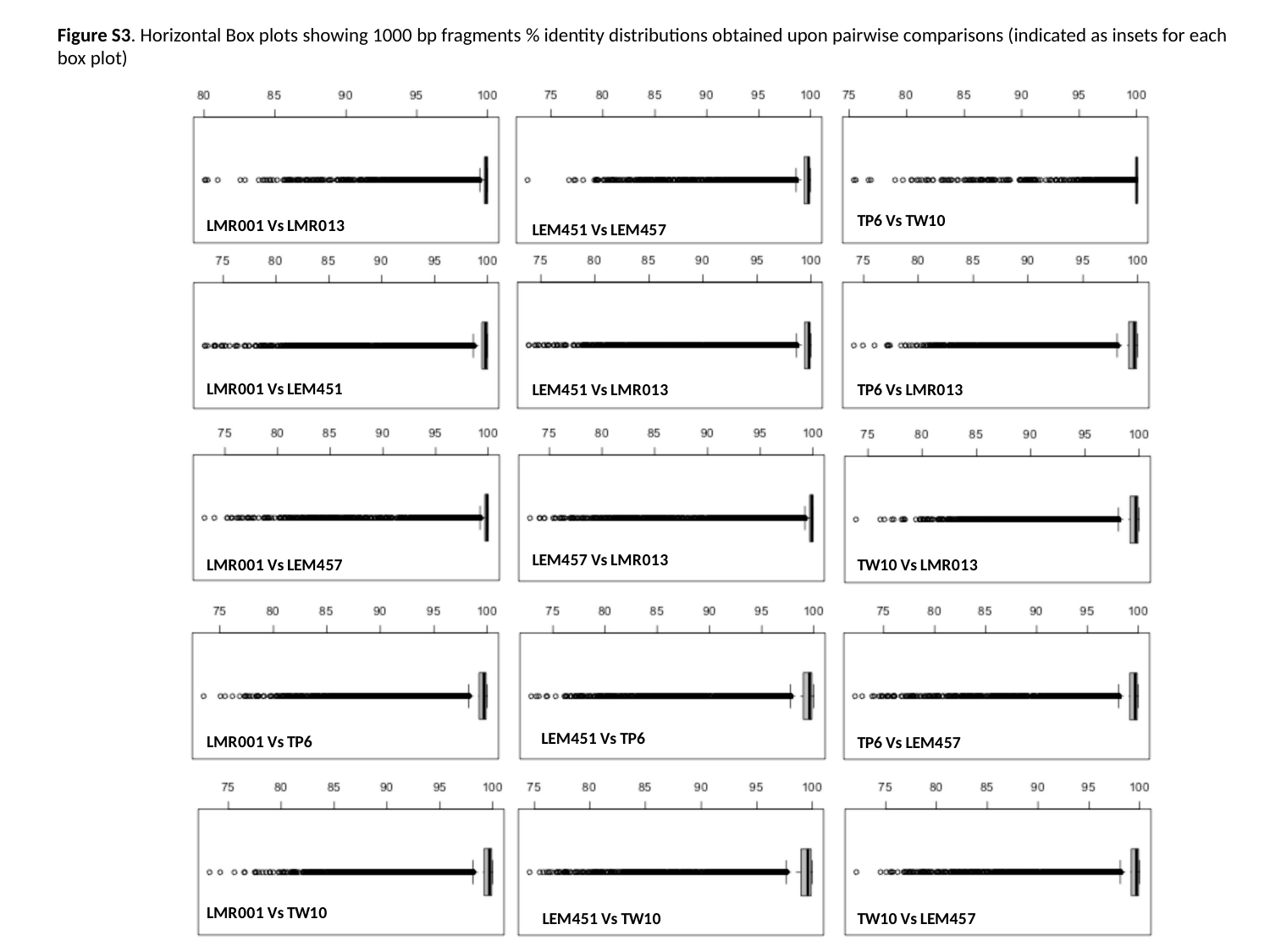

Figure S3. Horizontal Box plots showing 1000 bp fragments % identity distributions obtained upon pairwise comparisons (indicated as insets for each box plot)

## Slide 5
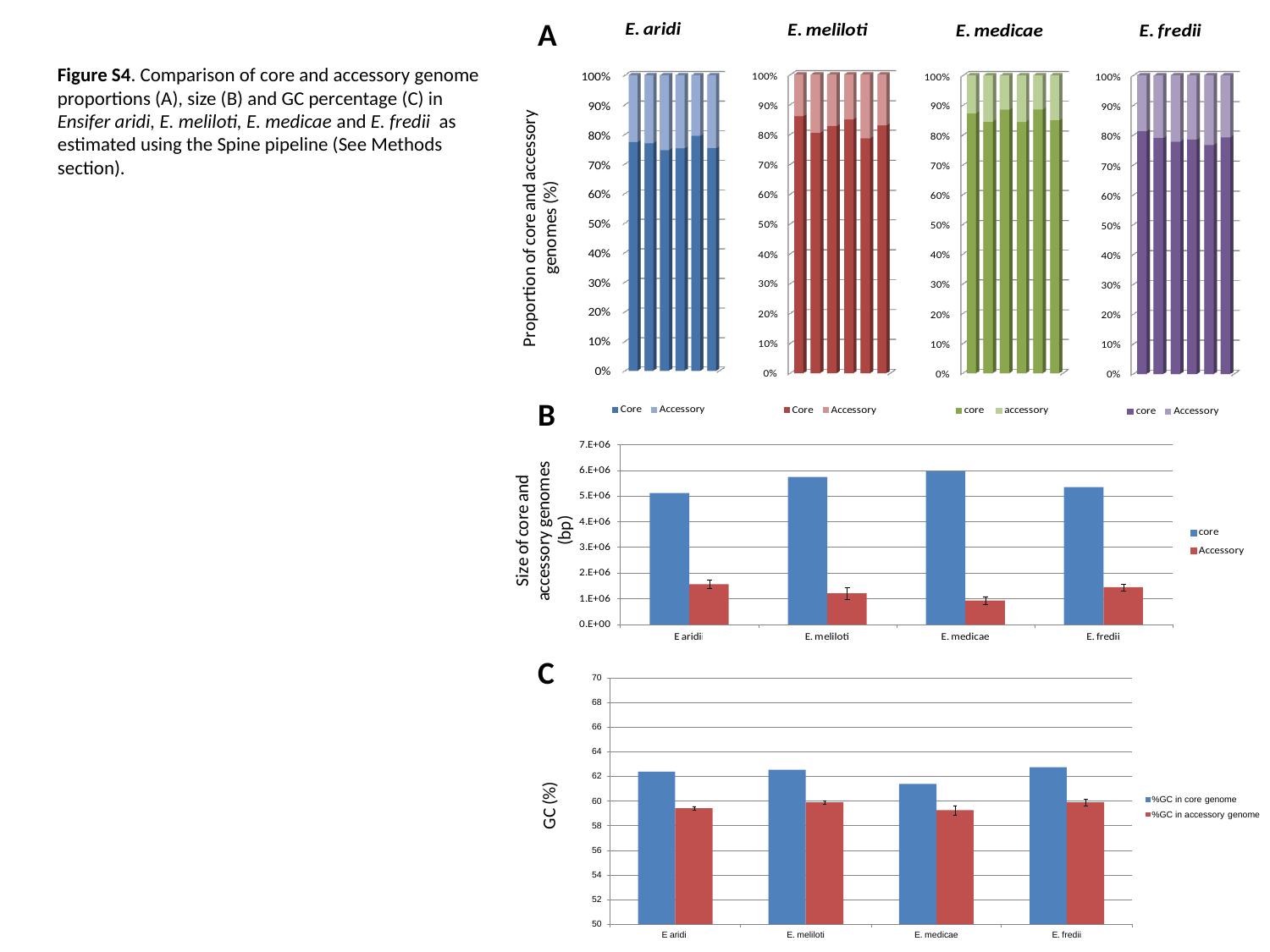

Figure S4. Comparison of core and accessory genome proportions (A), size (B) and GC percentage (C) in Ensifer aridi, E. meliloti, E. medicae and E. fredii as estimated using the Spine pipeline (See Methods section).

## Slide 6
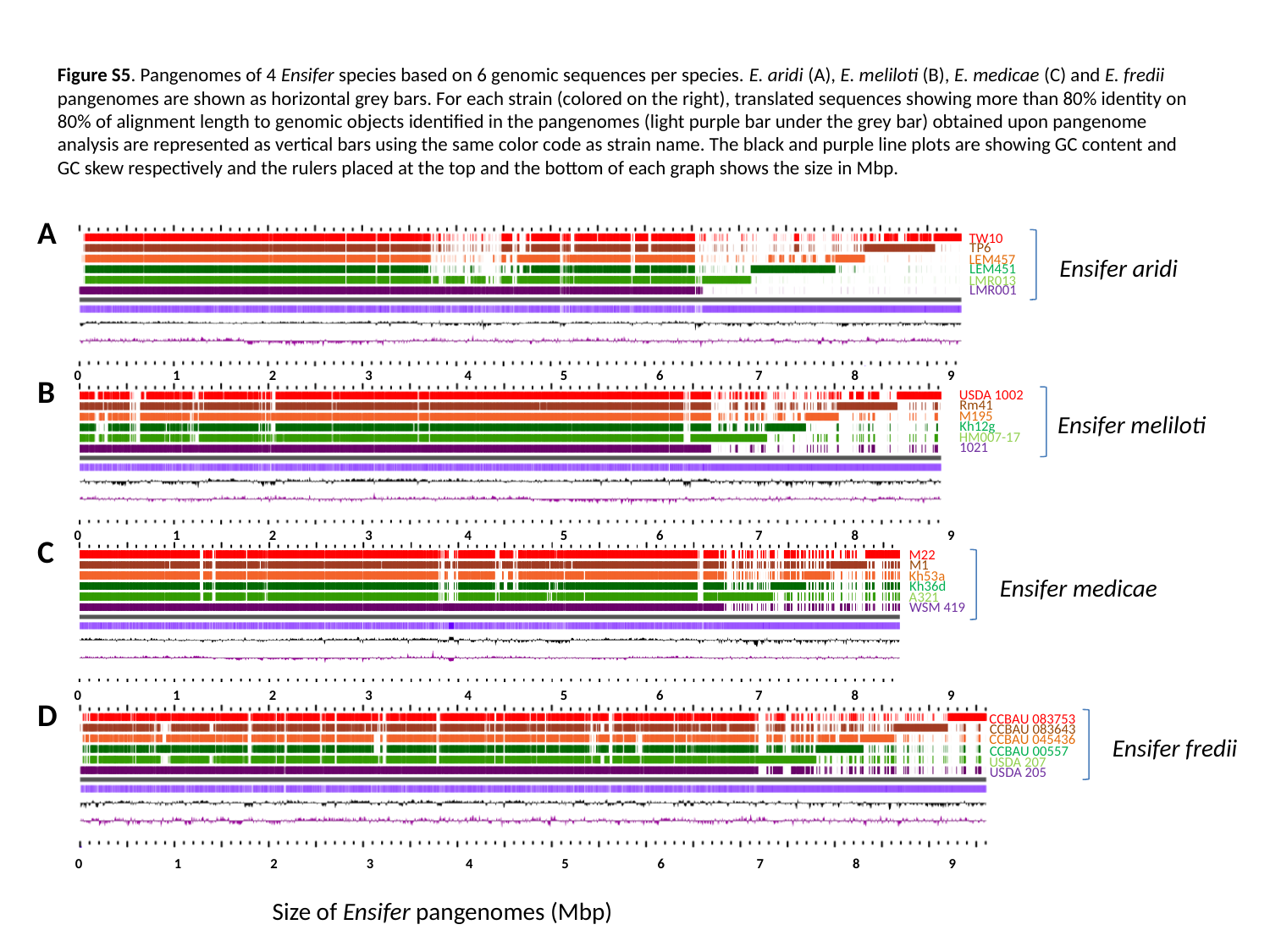

Figure S5. Pangenomes of 4 Ensifer species based on 6 genomic sequences per species. E. aridi (A), E. meliloti (B), E. medicae (C) and E. fredii pangenomes are shown as horizontal grey bars. For each strain (colored on the right), translated sequences showing more than 80% identity on 80% of alignment length to genomic objects identified in the pangenomes (light purple bar under the grey bar) obtained upon pangenome analysis are represented as vertical bars using the same color code as strain name. The black and purple line plots are showing GC content and GC skew respectively and the rulers placed at the top and the bottom of each graph shows the size in Mbp.
A
0 1 2 3 4 5 6 7 8 9
0 1 2 3 4 5 6 7 8 9
0 1 2 3 4 5 6 7 8 9
0 1 2 3 4 5 6 7 8 9
TW10
TP6
LEM457
Ensifer aridi
LEM451
LMR013
LMR001
B
USDA 1002
Rm41
M195
Ensifer meliloti
Kh12g
HM007-17
1021
C
M22
M1
Kh53a
Ensifer medicae
Kh36d
A321
WSM 419
D
CCBAU 083753
CCBAU 083643
CCBAU 045436
Ensifer fredii
CCBAU 00557
USDA 207
USDA 205
Size of Ensifer pangenomes (Mbp)

## Slide 7
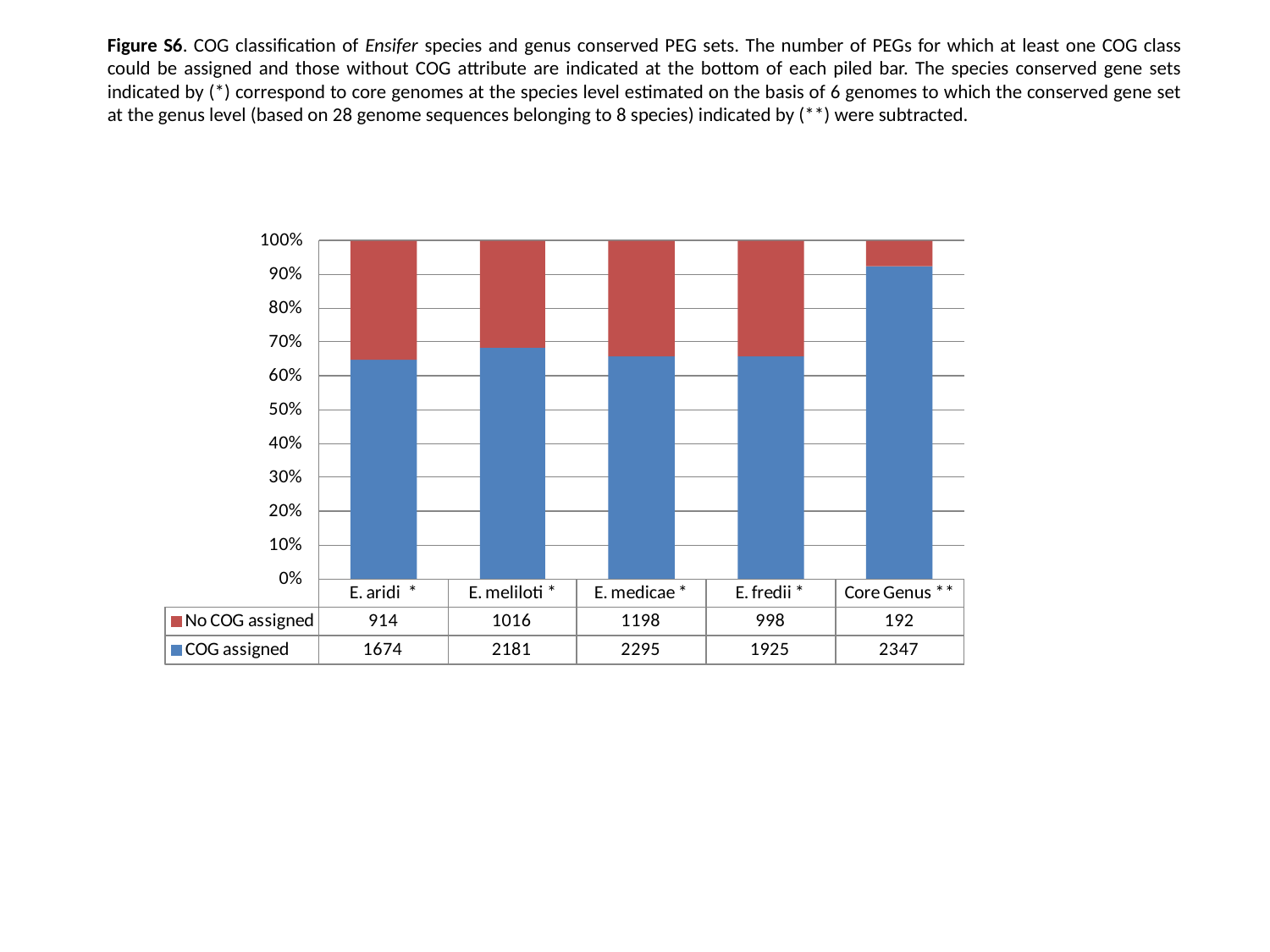

Figure S6. COG classification of Ensifer species and genus conserved PEG sets. The number of PEGs for which at least one COG class could be assigned and those without COG attribute are indicated at the bottom of each piled bar. The species conserved gene sets indicated by (*) correspond to core genomes at the species level estimated on the basis of 6 genomes to which the conserved gene set at the genus level (based on 28 genome sequences belonging to 8 species) indicated by (**) were subtracted.

## Slide 8
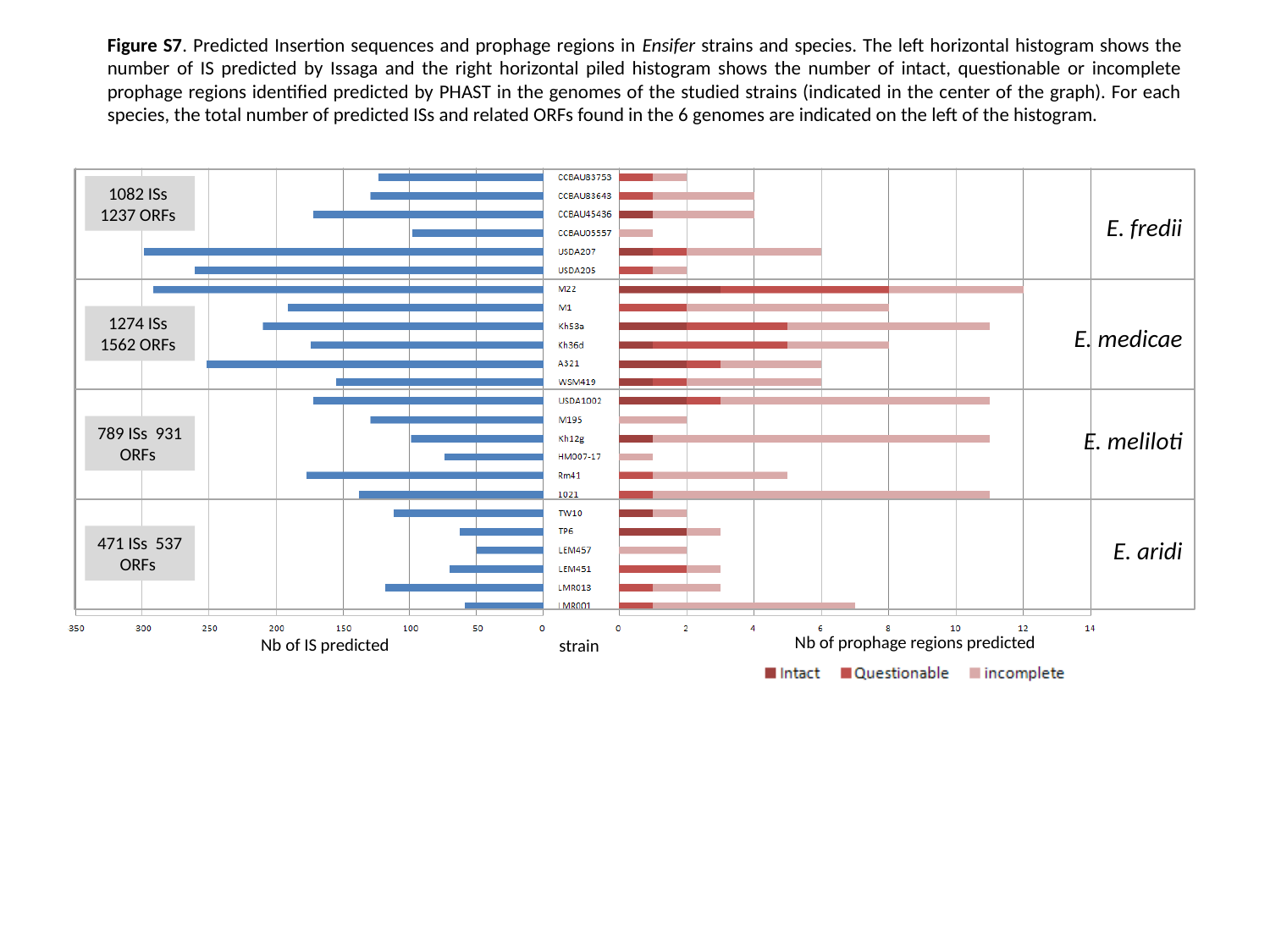

Figure S7. Predicted Insertion sequences and prophage regions in Ensifer strains and species. The left horizontal histogram shows the number of IS predicted by Issaga and the right horizontal piled histogram shows the number of intact, questionable or incomplete prophage regions identified predicted by PHAST in the genomes of the studied strains (indicated in the center of the graph). For each species, the total number of predicted ISs and related ORFs found in the 6 genomes are indicated on the left of the histogram.
E. fredii
E. medicae
E. meliloti
E. aridi
Nb of prophage regions predicted
Nb of IS predicted
1082 ISs 1237 ORFs
1274 ISs 1562 ORFs
789 ISs 931 ORFs
471 ISs 537 ORFs
strain

## Slide 9
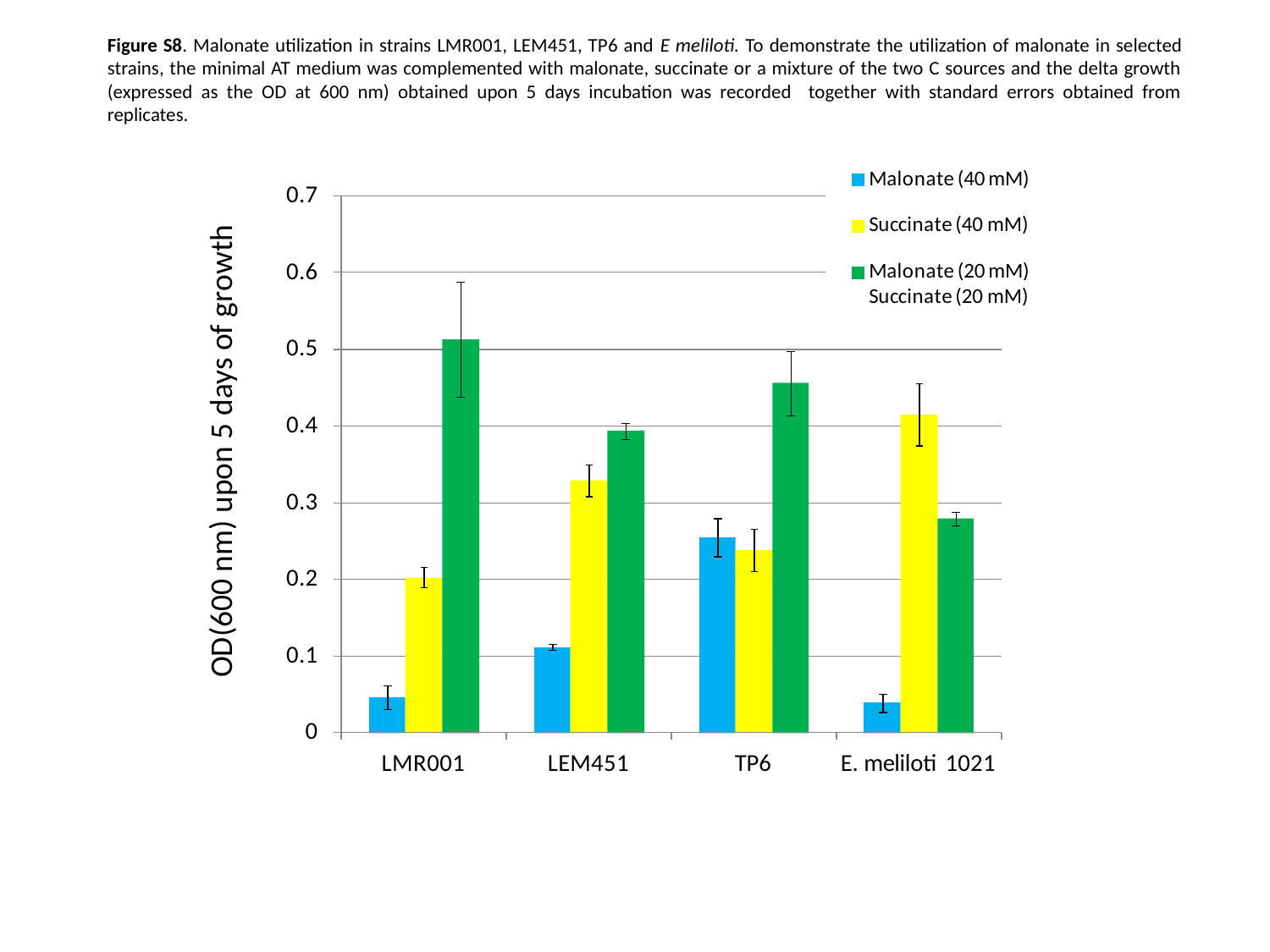

Figure S8. Malonate utilization in strains LMR001, LEM451, TP6 and E meliloti. To demonstrate the utilization of malonate in selected strains, the minimal AT medium was complemented with malonate, succinate or a mixture of the two C sources and the delta growth (expressed as the OD at 600 nm) obtained upon 5 days incubation was recorded together with standard errors obtained from replicates.
OD(600 nm) upon 5 days of growth
