# Supplementary figures and images for: Genomic characterization of Ensifer aridi, a proposed new species of nitrogen-fixing rhizobium recovered from Asian, African and American deserts
Source: BMC Genomics. 2017 Jan 14;18:85. doi: 10.1186/s12864-016-3447-y (PMC5237526; doi:10.1186/s12864-016-3447-y)

## Slide 1
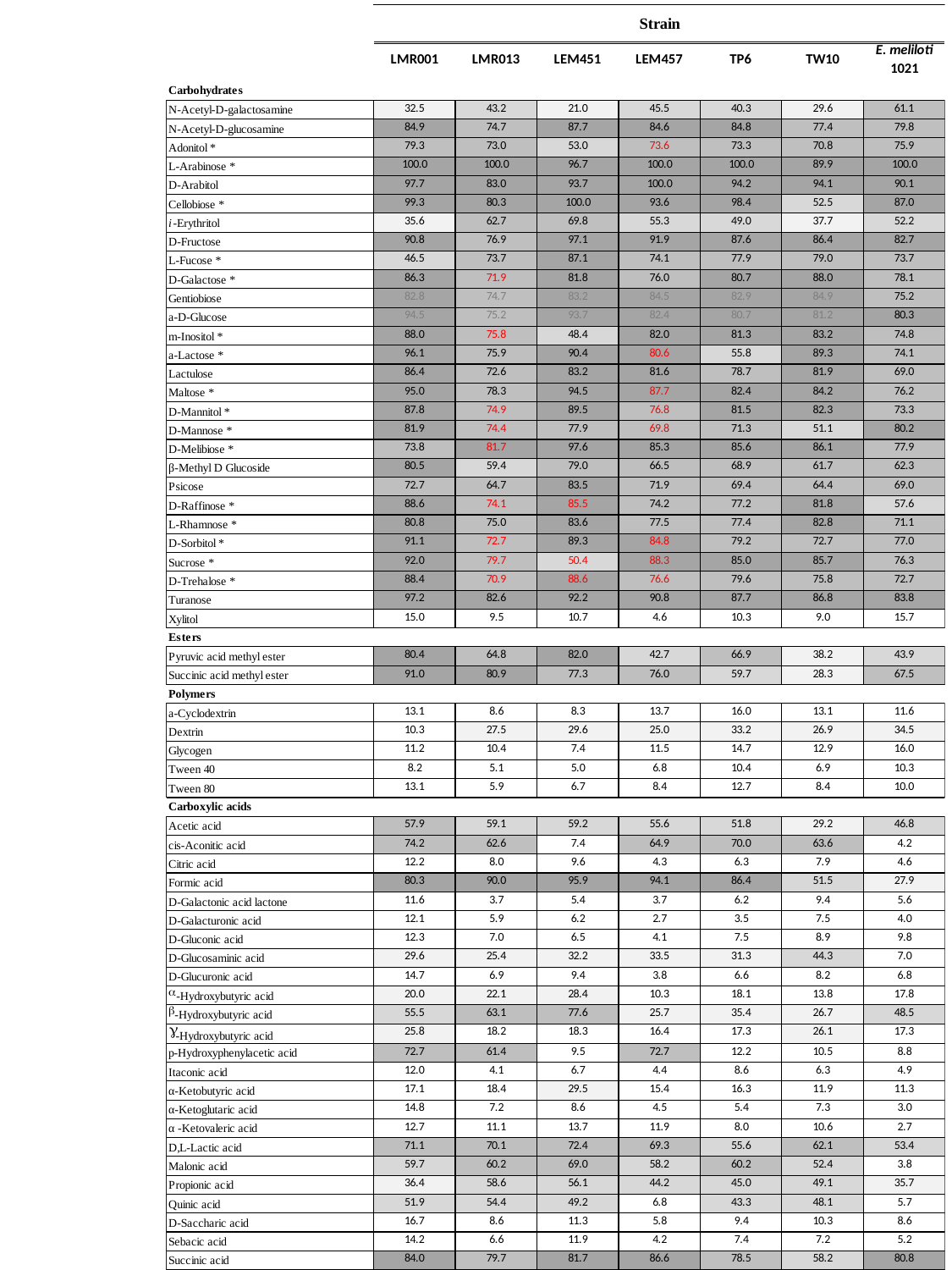

## Slide 2
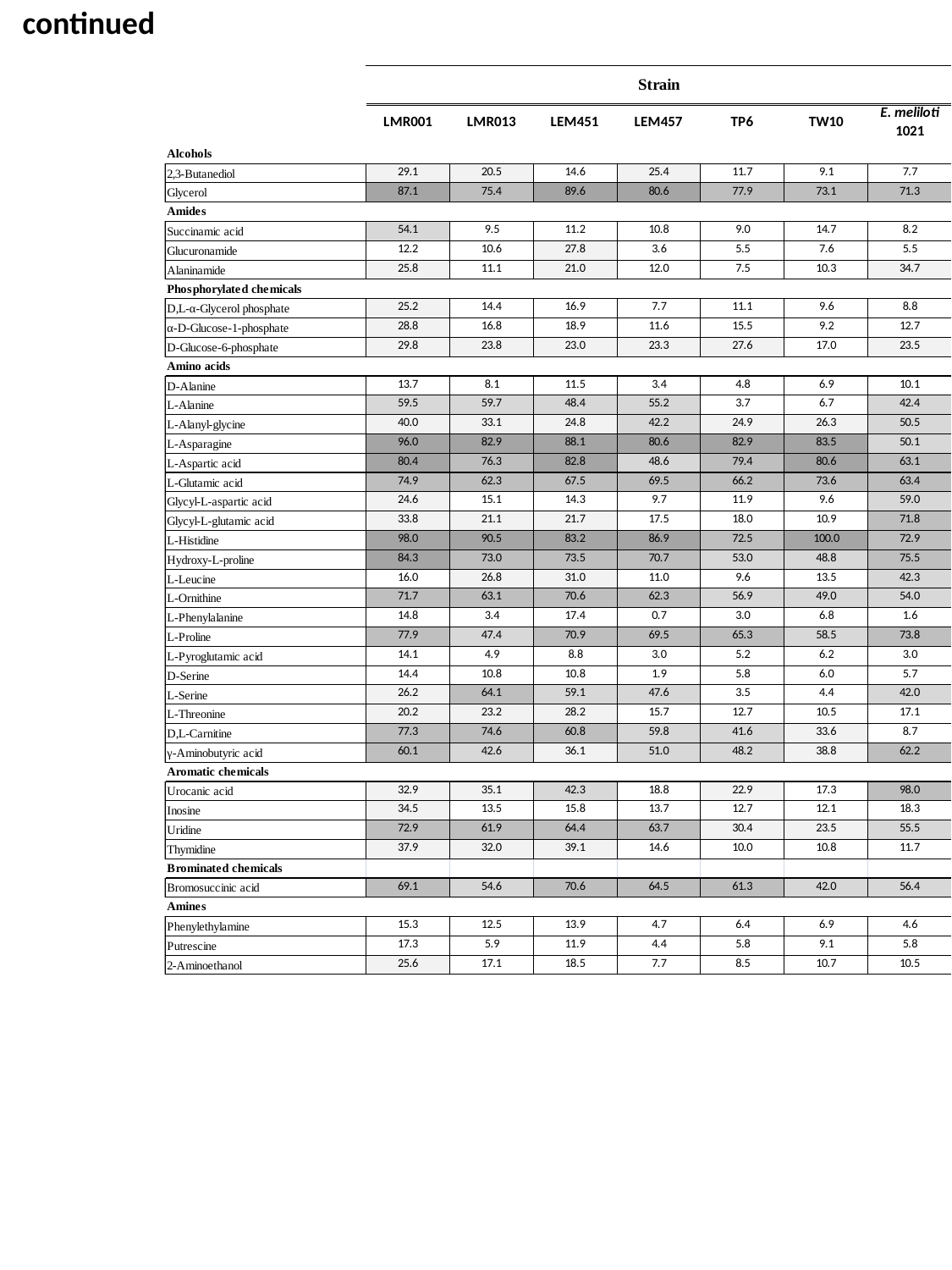

continued

Supplement: Additional file 3: — Ability of the new species to metabolize carbon sources in Biolog GN2 microplates compared to references strains E. meliloti 1021. For each strain, the data represent the mean of two replicates and is expressed as the % to maximum activity. The color code strengthen the relative activity of each carbon source ranging from no activity (white when <20% of maximal activity), and 4 levels of grey which correspond to ranges from weak activity (light grey) to high activities (dark grey) using respectively intervals ≤40, ≤60, ≤80 and ≤100%. Carbohydrates fermentation using the HiMedia disc assay are indicated (*) and red numbers indicate no fermentation activity using this method. (PPTX 121 kb) [file 12864_2016_3447_MOESM3_ESM.pptx]
